# Causality Verification for the Correlation between the Presence of Nonstarter Bacteria and Flavor Characteristics in Soft-Type Ripened Cheeses

**DOI:** 10.1128/spectrum.02894-22

**Published:** 2022-11-10

**Authors:** Ryosuke Unno, Toshihiro Suzuki, Yumika Osaki, Minenosuke Matsutani, Morio Ishikawa

**Affiliations:** a Department of Fermentation Science, Faculty of Applied Bioscience, Tokyo University of Agriculture, Tokyo, Japan; b NODAI Genome Research Center, Tokyo University of Agriculture, Tokyo, Japan; University of Minnesota

**Keywords:** bacteria, cheese, fermented food, flavor, multiomics, proteobacteria, ripening

## Abstract

Flavor characteristics of ripened cheese are established by various bacteria, such as lactic acid bacteria, *Actinobacteria*, and *Proteobacteria*, which spontaneously develop during the cheese-manufacturing process. We previously revealed the relationship between bacterial microbiota and flavor components in soft-type ripened cheeses by using a multiomics approach that combined metagenomics and metabolomics; however, we could not establish a causal relationship. This study aimed to substantiate the causal nature of the correlations revealed by the multiomics approach by using cheese-ripening tests with single isolate inoculation. The bacterial diversity and composition in surface mold-ripened cheeses from Japan and France varied, depending on the differences between the milks (pasteurized or raw), cheese positions (core or rind), and manufacturers. Although the volatile compounds did not clearly reflect the distinctive characteristics of the cheese samples, nonstarter lactic acid bacteria, *Actinobacteria*, and *Proteobacteria* positively correlated with ketones and sulfur compounds, as evidenced by a Spearman’s correlation analysis. Cheese-ripening tests conducted after inoculation with single bacterial strains belonging to the above-mentioned taxa confirmed that these bacteria formed volatile compounds, in agreement with the correlations observed. In particular, various flavor compounds, such as acids, esters, ketones, and sulfur compounds, were detected in cheese inoculated with *Pseudoalteromonas* sp. TS-4-4 strain. These findings provide important insights into the role of nonstarter bacteria in the development of cheese flavor and into the effectiveness of the multiomics approach in screening for bacteria that can improve the quality of cheese products.

**IMPORTANCE** Our previous study revealed that the existence of various bacteria, such as lactic acid bacteria, *Actinobacteria*, and *Proteobacteria*, clearly correlated with the abundance of flavor components, such as volatile compounds, in soft-type ripened cheeses via a multiomics approach that used 16S rRNA gene amplicon sequencing and headspace gas chromatography-mass spectrometry. However, this approach only showed correlations derived from statistical analyses rather than causal relationships. Therefore, in the present study, we performed cheese-ripening tests using nonstarter bacteria to substantiate the correlations revealed by the multiomics approach in soft-type ripened cheese. Our results suggest the capability of nonstarter bacteria, such as *Proteobacteria*, to impart flavor to cheese and the effectiveness of the multiomics approach in screening for microbial isolates that can improve the quality of cheese. Overall, our research provides new insights into the importance of bacteria in cheese production.

## INTRODUCTION

Cheese is a traditional fermented dairy food, with more than 1,000 varieties of distinctive appearance and flavor (1, 2). Various microorganisms originate from different sources, such as inoculum, raw materials, and cheese-making environments, settle into cheeses, and grow during the manufacturing process (3, 4). In general, starter lactic acid bacteria are deliberately inoculated and produce acids to coagulate milk, whereas nonstarter bacteria spontaneously develop during ripening and form flavor compounds (2). The diversity of nonstarter bacteria is the key factor for developing the characteristics of cheese (3). For example, cheeses that are processed by traditional methods, such as via the application of raw milk, possess rich and intense flavors because of a high microbial diversity (1, 3). On the other hand, industrial cheeses produced through pasteurization reduce the risk of foodborne illnesses to humans but simultaneously have less flavor than traditional cheeses because of their low microbial diversity (3, 5, 6). Thus, to safely and consistently produce cheese with unique sensory characteristics, it is necessary to understand the flavor-forming capabilities of various nonstarter bacteria that may be used as adjunct cultures.

In our previous research, we applied a multiomics approach that combined metagenomics and metabolomics to clarify the relationship between microorganisms and components in soft-type ripened cheeses (7). This approach revealed that the presence of bacteria, such as lactic acid bacteria (LAB), *Actinobacteria*, and marine-origin *Proteobacteria*, clearly correlated with the abundance of components, such as organic acids, free amino acids, and volatile compounds, in the cheeses. However, the relationships represent statistical correlations, not causal relationships. While the multiomics method is an effective approach by which to capture the candidates that improve cheese flavor and taste from an infinite number of microorganisms (8, 9), its effectiveness can be evaluated by verification using actual microorganisms. Therefore, our objective in this study was to verify the causality of such correlations between bacteria and components in ripened cheese that were revealed via the multiomics approach.

To this end, two issues needed to be resolved: one regarding the category of cheese samples, and the other about the resolution of taxonomic analysis via amplicon sequencing. Our previous research clearly showed correlations between bacterial microbiota and cheese components; however, we used samples from distinctively different categories, such as surface-mold ripened cheeses and bacterial smear-ripened cheeses (7). Hence, it was still unknown whether similar results would be obtained when cheese samples from the same category were used. Among various categories of cheese, surface-mold ripened cheese is popular with consumers and is produced worldwide (10). Although the cheeses in this category utilized similar manufacturing methods using Penicillium camemberti, the flavors of the final products differ according to the varieties of the cheeses. Therefore, to verify the statistical relationship between the bacteria and the flavor among cheeses in the same category, this study used surface-mold ripened cheeses from Japan and France.

Regarding the second issue, the 16S rRNA gene amplicon sequencing method used in previous research proved to be a powerful tool with which to provide comprehensive insights into complex bacterial microbiota in a sample (8, 11). Meanwhile, this method has a limitation in species-level or strain-level identification, owing to the short sequence lengths and highly conserved regions of the sequences under analysis (12). However, our previous research revealed clear correlations between the existence of specific bacteria and the abundance of specific compounds in the cheese, despite the validation of bacteria at the genus-level (7). Moreover, these correlations were clearly demonstrated, regardless of the type of cheese. In order to substantiate such correlations as causal relations, it was necessary to conduct a cheese-ripening test using bacterial strains that matched at the genus level but differed in the isolation source (e.g., categories of cheese or environment).

Thus, this study attempted to substantiate correlations revealed by a multiomics approach as causal relations. First, a multiomics approach revealed correlations between bacteria and components in surface-mold ripened cheese from Japan and France that belong to the same category. Bacterial microbiota and volatile compounds, components that contribute to flavor, were analyzed via 16S rRNA gene amplicon sequencing and headspace gas chromatography-mass spectrometry (HS-GC/MS), respectively. Thereafter, we selected the bacteria that were strongly correlated with volatile compounds and performed a cheese-ripening test using these bacterial strains. Eventually, we verified the consistency between the statistical correlations and the cheese-ripening test, and we evaluated the flavor-forming capabilities of nonstarter bacteria.

## RESULTS

### Physicochemical properties of cheese samples.

The product information, NaCl concentration, and pH of surface mold-ripened cheese samples are presented in Table 1. The salinity was approximately 1.0% in all samples, regardless of whether they were extracted from the core or rind. The pH of the rind (6.8 to 7.8) was higher than that of the core (5.7 to 7.0). Thus, the rind was considered to have slightly alkaline conditions.

**TABLE 1:** Product information and physicochemical characteristics of surface mold-ripened cheese samples^*a*^

Sample	Country	Material	diam (cm) × thickness (cm), wt (g)	NaCl (%)				pH			
				Core		Rind		Core		Rind	
				Avg	SE	Avg	SE	Avg	SE	Avg	SE
A1	Japan	Pasteurized cow’s milk	9.0 × 2.5, 120	1.0	0.03	1.0	0.03	5.8	0.03	6.8	0.00
A2	Japan	Pasteurized cow’s milk	12 × 3.0, 250	1.1	0.03	1.0	0.00	6.6	0.03	7.8	0.00
A3	Japan	Pasteurized cow’s milk	30 × 3.5, 2,000	0.9	0.00	0.9	0.03	5.7	0.00	7.1	0.00
B1	Japan	Raw cow’s milk	11 × 3.0, 250	0.8	0.03	0.8	0.00	5.8	0.03	7.3	0.00
B2	Japan	Raw cow’s milk	11 × 3.0, 250	1.0	0.00	1.0	0.03	5.6	0.00	7.4	0.00
C	France	Raw cow’s milk	36 × 3.0, 2,500	1.1	0.03	1.1	0.00	7.0	0.03	7.7	0.00
D	France	Raw cow’s milk	24 × 3.5, 1,500	0.9	0.03	0.8	0.00	6.6	0.03	7.0	0.03

a Each sample was divided into rind and core before measurement. Values were calculated from three cheese types.

### Bacterial community in surface mold-ripened cheese.

**FIG 1:**
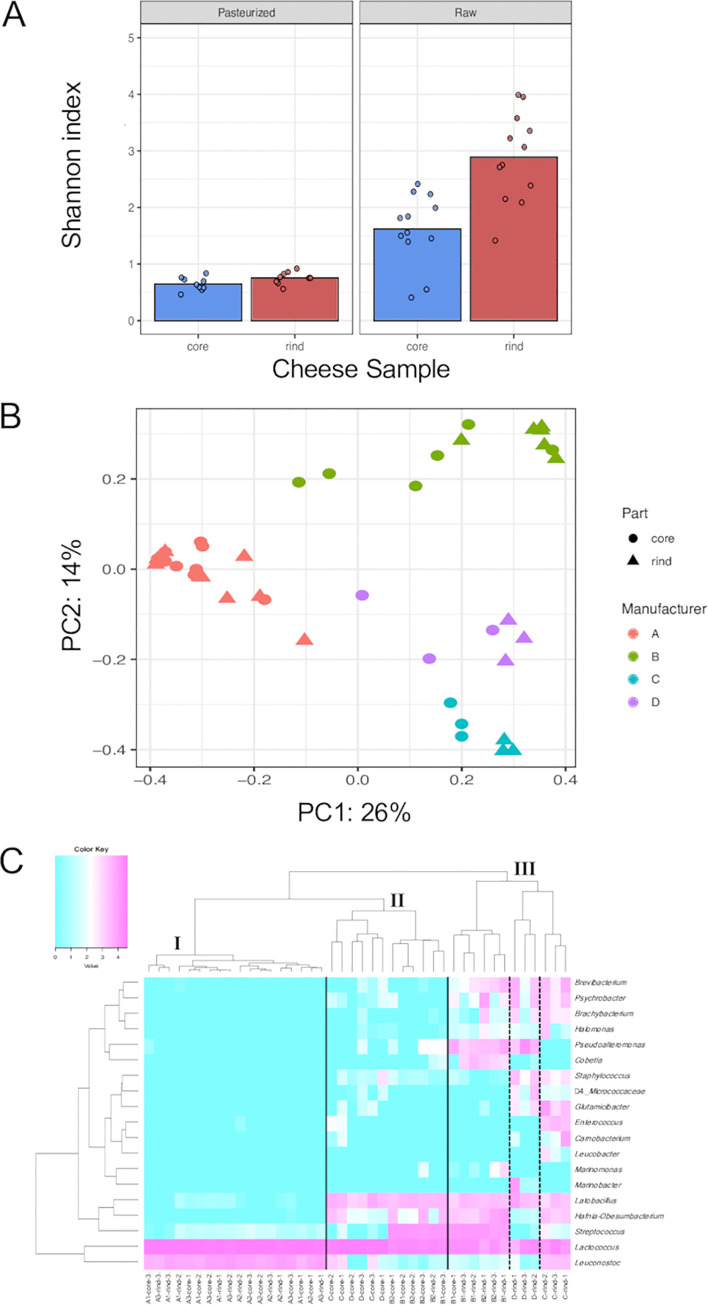
Bacterial community structure in surface mold-ripened cheese samples. (A) Comparison of cheese samples in terms of alpha diversity via the Shannon index. Plots are shown separately for the differences of milk processing methods and cheese sampling positions. (B) Jaccard distance principal coordinates analysis. The plots are colored to denote the differences in manufacturers (cheeses A, B, C, and D). The circular and triangular plots indicate the core and rind, respectively. (C) Hierarchically clustered heat map, based on the relative abundance of the bacterial genera. Data are clustered using the complete linkage method with the Euclidean distance. Only bacteria with a relative abundance of >0.5% in at least one sample are shown. Purple indicates high occupancy, and light blue indicates low or nonexistent occupancy. Cheese sample clusters are represented by I, II, and III.

A total of 8,052,836 reads were generated by the 16S rRNA gene amplicon sequencing of 42 cheese samples. After the quality control of the sequencing reads, 40,000 reads per sample were subsampled without replacement and used for diversity and hierarchical analyses (Fig. 1; Table S1). The alpha diversity via the Shannon index (13) was significantly higher in the samples obtained from raw cow’s milk cheese (cheeses B, C, and D) than that observed in the pasteurized cow’s milk cheese samples (cheese A) (Kruskal-Wallis test, *P* < 0.05) (Fig. 1A). Among raw cow’s milk cheeses, the rind samples showed higher alpha diversity via the Shannon index than did the core samples (Kruskal-Wallis test, *P* < 0.05). Other alpha diversity metrices (Feature abundance [14] and Pielou's evenness [15]) also showed similar results (Kruskal-Wallis test, *P* < 0.05, data not shown). Further, a principal coordinates analysis (PCoA) based on the Jaccard distance (16) revealed the difference in bacterial communities among manufacturers (permutational analysis of variance [PERMANOVA] test, *P* < 0.01) (Fig. 1B). The Jaccard index does not consider the abundance of species but the presence or absence of species. Hence, this suggested that the differences in the qualitative composition of bacteria in the cheeses were related to the differences in manufacturers.

To further clarify the bacterial composition, a heat map was created to show the degree of bacterial occupancy in the cheese samples (Fig. 1C). Bacteria that showed a relative abundance of >0.5% at the genus level in at least one sample were subjected to a hierarchical clustering analysis. The cheese samples were divided into three major clusters (I, II, and III) via hierarchical clustering. Cluster I was composed of cheese A. Cluster II was comprised of the cores of cheeses B, C, and D, while cluster III was comprised of the rinds of these cheeses. The dominant phyla in the bacterial communities of the cheese samples were *Firmicutes*, *Actinobacteria*, and *Proteobacteria*. Clusters I and II were predominantly comprised of LAB. At the genus level, cluster I was dominated by *Lactococcus* and *Leuconostoc*, whereas cluster II was dominated by *Lactococcus* and *Lactobacillus*. Additionally, Streptococcus and *Leuconostoc* were detected in cheeses B and C, respectively. The genus *Hafnia*-*Obesumbacterium*, which belongs to the phylum *Proteobacteria*, was also detected in cheeses B and C.

The bacterial community of cluster III was more diverse than that of clusters I and II. In addition to the bacteria detected in cluster II, cluster III included *Brevibacterium* and *Brachybacterium*, which belong to the phylum *Actinobacteria*, and *Psychrobacter* and *Halomonas*, belonging to the phylum *Proteobacteria*. Furthermore, cluster III formed a subcluster, owing to the differences in the cheese manufacturers. While *Pseudoalteromonas*, *Cobetia*, and *Marinomonas* (*Proteobacteria*) were detected in cheese B from Japan, Staphylococcus (*Firmicutes*) and *Glutamicibacter* (*Actinobacteria*) were detected in cheeses C and D from France. In the samples of the French cheeses, *Enterococcus* and *Carnobacterium*, known as nonstarter LAB (NSLAB), and *Leucobacter*, which belong to the phylum *Actinobacteria*, were detected in cheese C. *Pseudoalteromonas* and *Marinobacter* (*Proteobacteria*) were detected in cheese D. Thus, the bacterial microbiota of surface mold-ripened cheese reflected the differences in the milks (pasteurized or raw), the locations from where the samples were extracted (core or rind), and the manufacturers.

### Relationship between bacteria and volatile compounds of surface mold-ripened cheese.

**FIG 2:**
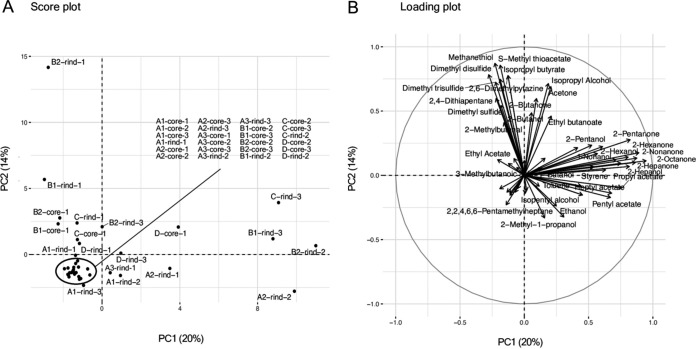
Principal components analysis, based on volatile compounds detected in surface mold-ripened cheese samples. (A) Score plot. (B) Loading plot.

We identified 59 volatile compounds in 42 cheese samples (Table S2) through HS-GC/MS and used them for a principal components analysis (PCA) (Fig. 2). The score plot showed the aggregation of many samples, such as the core samples of cheese A (Fig. 2A). Moreover, the loading plot showed that most ketones were distributed in the positive direction on the first principal component (PC1) axis, which explained 20% of the total variance. The sulfur compounds were distributed in the positive direction on the second principal component (PC2) axis, which explained 14% of the total variance (Fig. 2B; Table S3). These results implied that the content of ketones and sulfur compounds characterized the 42 cheese samples used in this study. However, as opposed to the bacterial microbiota analysis, the PCA did not show a clear tendency based on the differences among cheeses, such as the pasteurization status of the milk, the location on the cheese from where the sample was extracted, and/or the manufacturers. Therefore, we verified whether the flavor characteristics of the cheese samples appeared to be associated with the microbiota by performing a Spearman’s correlation analysis between the bacterial microbiota and the volatile compounds.

**FIG 3:**
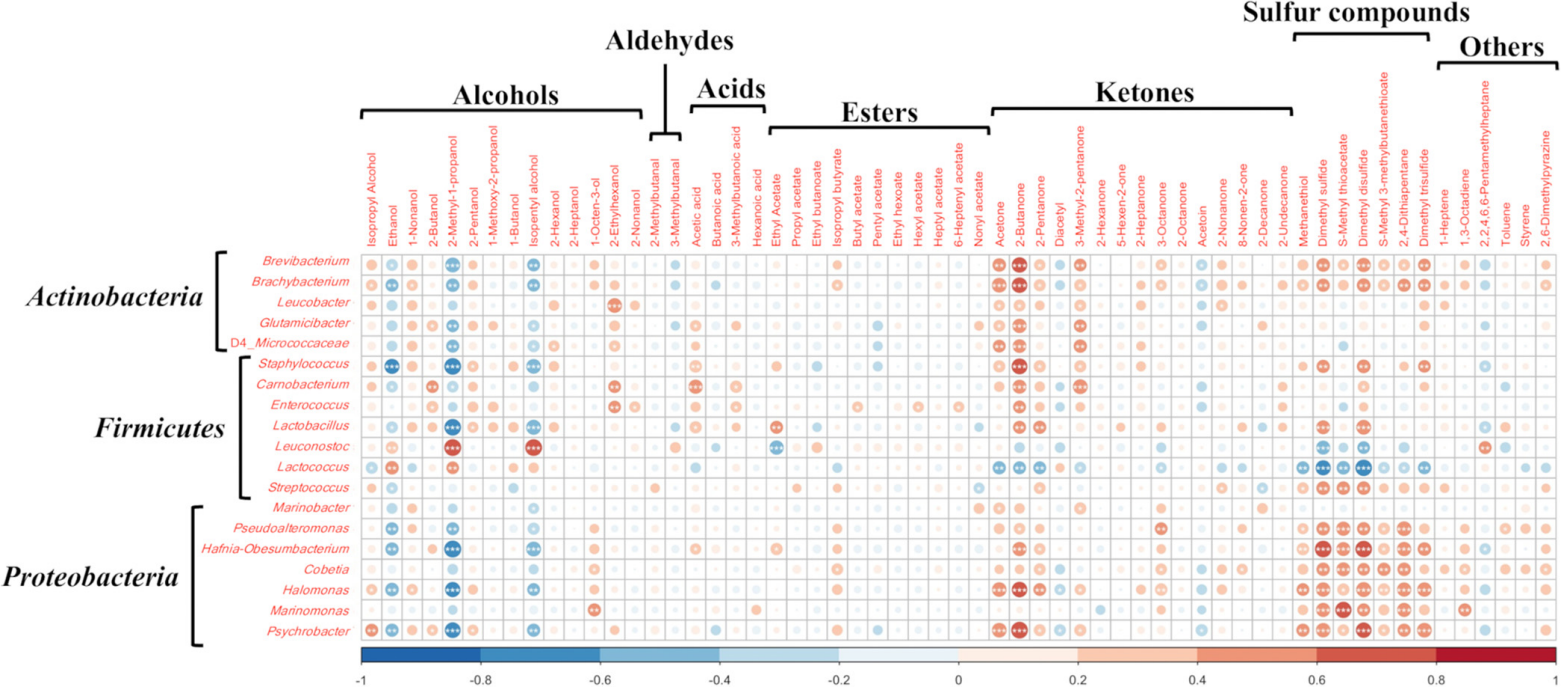
Correlations between bacterial genera and volatile compounds detected in surface mold-ripened cheese samples. The number of stars indicates the significance level. *, *P* < 0.05; **, *P* < 0.01; ***, *P* < 0.001.

The correlation analysis revealed a relationship between specific bacteria and flavor compounds. The degree of correlation is shown in the heat map of Fig. 3. In phylum *Firmicutes*, *Lactococcus* and *Leuconostoc* positively correlated with alcohols, such as ethanol, 2-methyl-1-propanol, and isopentyl alcohol. Meanwhile, Streptococcus positively correlated with sulfur compounds, such as dimethyl disulfide and S-methyl thioacetate. Additionally, other genera in *Firmicutes* positively correlated with 2-butanone. *Actinobacteria* positively correlated with ketones, such as acetone, 2-butanone, and 3-methyl-2-pentanone. Within the phylum *Actinobacteria*, *Brevibacterium* and *Brachybacterium* positively correlated with sulfur compounds, such as dimethyl sulfide and dimethyl disulfide. Genera within the phylum *Proteobacteria*, except for *Marinobacter*, showed strong positive correlations with sulfur compounds, such as methanethiol, S-methyl thioacetate, and dimethyl disulfide. *Halomonas* and *Psychrobacter* also positively correlated with 2-butanone and acetone. These results showed that besides LAB, nonstarter bacteria, such as *Actinobacteria* and *Proteobacteria*, were involved in producing specific volatile compounds in cheese.

### Succession of bacterial growth and surface appearance in cheese-ripening tests.

**FIG 4:**
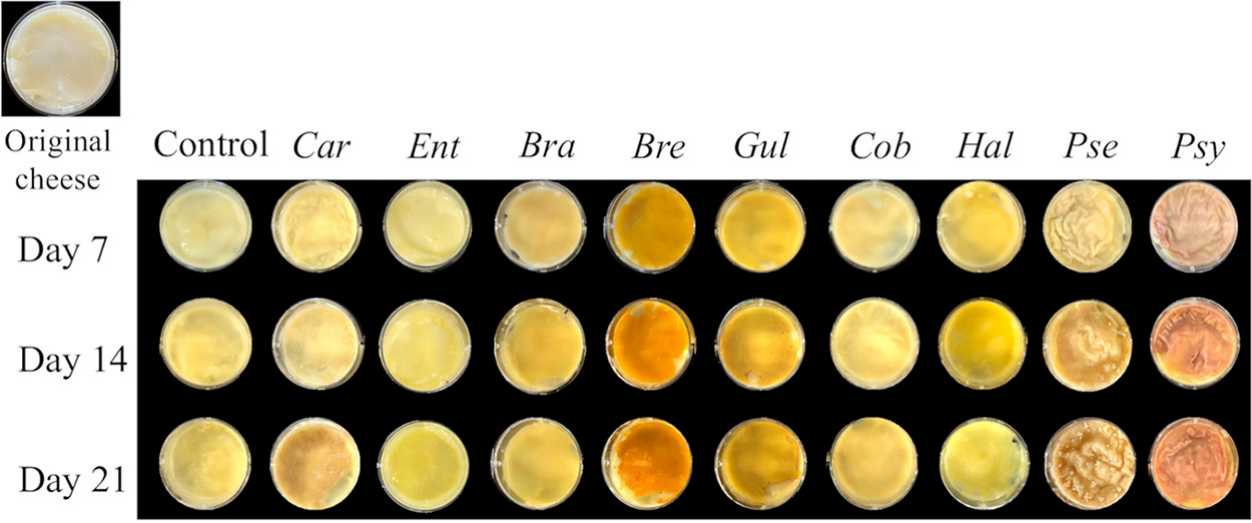
Change in the color of the cheese surface inoculated with bacterial strains during ripening. *Car*, *Carnobacterium* sp. SN-1-4; *Ent*, *Enterococcus* sp. 8B5; *Bra*, *Brachybacterium* sp. FU-4-2; *Bre*, *Brevibacterium* sp. FU-2-6, *Gul*, *Gultamicibacter* sp. FU-2-5; *Cob*, Cobetia marina NBRC 102605; *Hal*, *Halomonas* sp. SN-2-8; *Pse*, *Pseudoalteromonas* sp. TS-4-4; *Psy*, *Psychrobacter* sp. FU-2-4.

To demonstrate the correlations between bacteria and flavor, bacterial strains were isolated from eight kinds of Japanese soft-type ripened cheeses using several media (Table S4). A total of 47 strains were identified at the genus-level using 16S rRNA gene sequencing (data not shown). Based on the bacterial correlation with volatile compounds in surface mold-ripened cheese, seven isolated strains, one of which was provided by the Biological Resource Center, NITE (NBRC) (17), and another of which was isolated from Brie cheese by Unno et al. (18) were selected and used for the cheese-ripening tests. The isolation source, medium, and results of a basic local alignment search tool (BLAST) search are summarized in Table 2. The viable bacterial counts (in colony forming units [CFU], as determined using the plate count technique) on days 0, 7, 14, and 21 of cheese-ripening are shown in Table 3. The control showed no microbial growth in TSB or in TSB + NaCl 3% agar medium during ripening. The log_10_ CFU/g in all samples inoculated with bacteria was between 7.0 and 10 on day 7. Although the CFU of *Pseudoalteromonas* sp. TS-4-4 decreased from day 7 and disappeared on day 21, the other strains survived until day 21. Some bacteria changed the color of the cheese surface (Fig. 4). In particular, cheeses inoculated with *Brevibacterium* sp. FU-2-6, *Pseudoalteromonas* sp. TS-4-4, and *Psychrobacter* sp. FU-2-4 turned orange, dark cream, and coral, respectively. These results showed that the tested strains could grow on cheese, with some strains affecting the color of the cheese surface.

**TABLE 2:** Identification and growth medium of bacterial strains used in cheese-ripening test

Strain name	Source	Reference	Isolation medium	Culture medium	Closest strain by BLAST search	Similarity % (bp analyzed)	Accession no.
*Carnobacterium* sp. SN-1-4	Japanese soft-type brine-washed, ripened cheese, from raw cow’s milk	Present study	Funke	TSB	Carnobacterium mobile DSM 4848	98.00 (1, 148)	LC685115
*Enterococcus* sp. 8B5	French soft-type ripened cheese (Brie de Meaux)	(18)		TSB	Enterococcus faecalis LMG 7937	99.80 (500)	LC507024
*Brachybacterium* sp. FU-4-2	Japanese soft-type brine-washed, ripened cheese, from raw cow’s milk	Present study	MB + Glucose	TSB + NaCl	Brachybacterium alimentarium CNRZ 925	99.72 (715)	LC685116
*Brevibacterium* sp. FU-2-6	Japanese soft-type brine-washed, ripened cheese, from raw cow’s milk	Present study	Funke + NaCl	TSB + NaCl	Brevibacterium aurantiacum NCDO 739	96.52 (1, 288)	LC685117
*Glutamicibacter* sp. FU-2-5	Japanese soft-type brine-washed, ripened cheese, from raw cow’s milk	Present study	Funke + NaCl	TSB + NaCl	Glutamicibacter bergerei Ca106	98.64 (1, 239)	LC685118
Cobetia marina NBRC 102605	Coastal sea water	(17)		TSB + NaCl			AB681880
*Halomonas* sp. SN-2-8	Japanese soft-type brine-washed, ripened cheese, from raw cow’s milk	Present study	PCAM + V + CV + NaCl	TSB + NaCl	Halomonas zhanjiangensis JSM 078169	99.1 (1, 219)	LC685119
*Pseudoalteromonas* sp. TS-4-4	Japanese soft-type brine-washed, ripened cheese, from raw cow’s milk	Present study	MB	TSB + NaCl	Pseudoalteromonas prydzensis MB8-11	97.92 (1, 007)	LC685120
*Psychrobacter* sp. FU-2-4	Japanese soft-type brine-washed, ripened cheese, from raw cow’s milk	Present study	Funke + NaCl	TSB + NaCl	Psychrobacter alimentarius JG-100	96.45 (1, 208)	LC685121

**TABLE 3:** Microbial counts during ripening in cheeses inoculated with different bacterial strains^*a*^

Strain	log_10_ CFU g^−1^							
	Day 0		Day 7		Day 14		Day 21	
	Avg	SE	Avg	SE	Avg	SE	Avg	SE
*Carnobacterium* sp. SN-1-4	6.66	0.03	9.60	0.15	9.46	0.11	9.40	0.39
*Enterococcus* sp. 8B5	5.34	0.11	9.56	0.05	9.62	0.08	9.35	0.03
*Brachybacterium* sp. FU-4-2	7.55	0.23	10.69	0.02	10.83	0.05	10.87	0.05
*Brevibacterium* sp. FU-2-6	5.73	0.10	10.92	0.09	11.26	0.02	11.15	0.02
*Glutamicibacter* sp. FU-2-5	7.36	0.01	10.67	0.03	10.78	0.08	10.69	0.05
Cobetia marina NBRC 102605	5.72	0.53	10.11	0.10	9.85	0.04	9.36	0.23
*Halomonas* sp. SN-2-8	7.06	0.25	10.05	0.07	10.29	0.02	9.87	0.09
*Pseudoalteromonas* sp. TS-4-4	5.96	0.24	7.55	0.53	1.67	1.67	0.00	0.00
*Psychrobacter* sp. FU-2-4	7.26	0.06	10.65	0.03	10.46	0.01	10.52	0.11

a Values were calculated from three cheeses that were subjected to the ripening test. The control (which contained Ringer’s solution instead of an inoculant) showed no microbial growth during ripening.

### Assessment of the flavor-forming ability of isolates through cheese-ripening tests.

**FIG 5:**
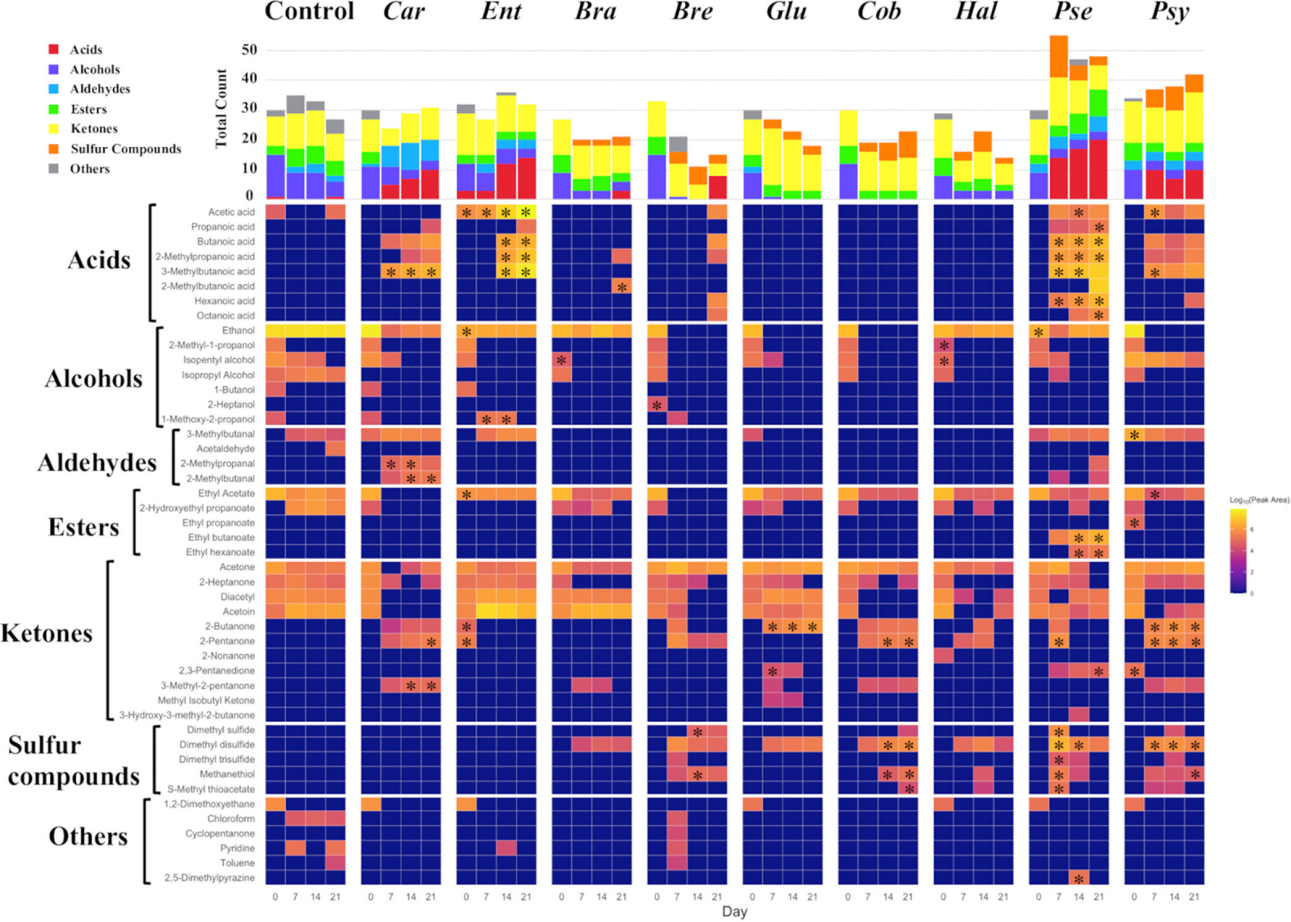
Fluctuation of volatile compounds in cheeses inoculated with bacterial strains during ripening. Heat map shows the average peak areas (*n* = 3) of volatile compounds detected on days 0, 7, 14, and 21. Yellow indicates a large peak area, while blue indicates a small or zero peak area. The bar plot at the top shows the changes in the total count of volatile compounds detected in all of the cheese samples that were inoculated with same bacterial strain (*n* = 3). A statistical test was used to compare the control and the samples that were inoculated with a bacterial strain. The asterisk indicates a statistically significant difference between the control and the bacterial inoculated samples (*P* < 0.05). *Car*, *Carnobacterium* sp. SN-1-4; *Ent*, *Enterococcus* sp. 8B5; *Bra*, *Brachybacterium* sp. FU-4-2; *Bre*, *Brevibacterium* sp. FU-2-6, *Gul*, *Gultamicibacter* sp. FU-2-5; *Cob*, Cobetia marina NBRC 102605; *Hal*, *Halomonas* sp. SN-2-8; *Pse*, *Pseudoalteromonas* sp. TS-4-4; *Psy*, *Psychrobacter* sp. FU-2-4.

HS-GC/MS detected 46 volatile compounds in the control and tested samples (Table S5). As opposed to the control cheese without bacterial inoculation, all samples inoculated with bacteria showed changes in the composition of volatile compounds during ripening (Fig. 5). Acids increased in cheeses inoculated with LAB strains *Carnobacterium* sp. SN-1-4 and *Enterococcus* sp. 8B5. In particular, the abundance of 3-methylbutanoic acid from the cheeses inoculated with *Carnobacterium* sp. SN-1-4 and the abundance of acetic acid, butanoic acid, 2-methylpropanoic acid, and 3-methylbutanoic acid from the cheeses inoculated with *Enterococcus* sp. 8B5 showed significant differences compared to a control. Moreover, the cheeses inoculated with *Carnobacterium* sp. SN-1-4 showed the presence of aldehydes, such as 2-methylpropanal and 2-methylbutanal, and ketones, such as 2-pentanone and 3-methyl-2-pentanone, after day 7 of ripening.

Sulfur compounds and ketones were detected during ripening in the cheese samples inoculated with *Actinobacteria*. Among them, four types of sulfur compounds, especially dimethyl disulfide and methanethiol, were detected in the cheeses inoculated with *Brevibacterium* sp. FU-2-6, whereas four types of ketones, especially 2-butanone and 2,3-pentanedione, were detected in the cheeses inoculated with *Glutamicibacter* sp. FU-2-5.

Similar to *Actinobacteria*, the inoculation with *Proteobacteria* caused the formation of various sulfur compounds and ketones. After day 7, 2-butanone, 2-pentanone, dimethyl disulfide, methanethiol, and S-methyl-thioacetate were detected in all four samples (inoculated with Cobetia marina NBRC 102605, *Halomonas* sp. SN-2-8, *Pseudoalteromonas* sp. TS-4-4, and *Psychrobacter* sp. FU-2-4). Furthermore, the cheeses inoculated with Cobetia marina NBRC 102605, *Pseudoalteromonas* sp. TS-4-4, and *Psychrobacter* sp. FU-2-4 showed significant differences in the presence of sulfur compounds, such as dimethyl disulfide and methanethiol, and ketones, such as 2-pentanone, compared to the control. From day 7 to day 21 of ripening, 8 and 5 types of acids were detected in cheeses inoculated with *Pseudoalteromonas* sp. TS-4-4 and *Psychrobacter* sp. FU-2-4, respectively. Additionally, *Pseudoalteromonas* sp. TS-4-4 produced esters, such as ethyl butanoate and ethyl hexanoate.

**FIG 6:**
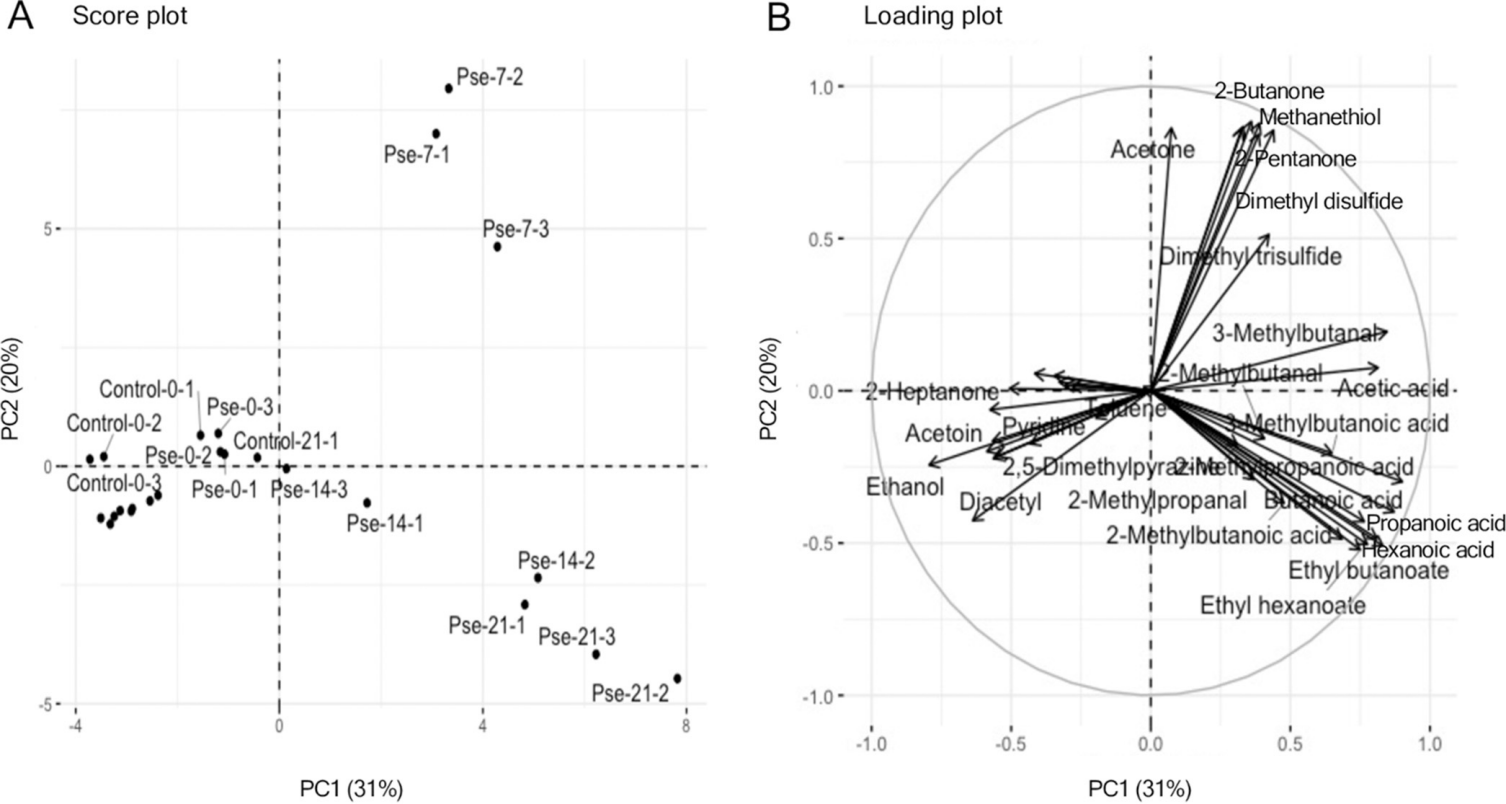
Principal components analysis, based on volatile compounds detected in cheeses inoculated with *Pseudoalteromonas* sp. TS-4-4 and controls. (A) Score plot. (B) Loading plot. The sample name separated by a hyphen indicates the control or bacterial name, the ripening days, and the sample number from the front. *Pse*, *Pseudoalteromonas* sp. TS-4-4.

Comparing the total number of volatile compounds detected in each tested cheese, the cheese inoculated with *Pseudoalteromonas* sp. TS-4-4 was the highest and remained at that level from day 7 to day 21 of ripening (Fig. 5). Moreover, the cheese inoculated with *Pseudoalteromonas* sp. TS-4-4 was significantly different from the control in terms of the presence of many components (acids, esters, ketones, and sulfur compounds). We subjected this cheese sample, along with the control sample, to a PCA to evaluate the effect of *Pseudoalteromonas* sp. TS-4-4 on cheese (Fig. 6). PC1, which explained 31% of the total variance, distinguished the samples inoculated with *Pseudoalteromonas* sp. TS-4-4 from the controls (Fig. 6A). Moreover, PC2, which explained 20% of the total variance, emphasized the difference of ripening days (day 7 and day 21). The loading plot confirmed that acetone, 2-butanone, 2-pentanone, and sulfur compounds contributed positively to PC2 and were associated with the *Pseudoalteromonas* sp. TS-4-4 samples on day 7 of ripening, whereas the esters and acids, such as ethyl butanoate, ethyl hexanoate, propanoic acid, and hexanoic acid, contributed negatively to PC2 and were associated with the samples on day 21 (Fig. 6B; Table S6).

These results revealed that the bacterial strains used in this study affected the composition of the volatile compounds (acids, esters, ketones, and sulfur compounds) in cheese. In addition, the ripening tests substantiated the correlations between the nonstarter bacteria and the volatile compounds, as shown by the multiomics approach.

## DISCUSSION

This study was performed to verify the causality of the correlations between the nonstarter bacteria and the volatile compounds in soft-type ripened cheese that were revealed via the multiomics approach. First, we verified the correlations between the nonstarter bacteria and the volatile compounds in surface-mold ripened cheeses by using a multiomics approach. Next, to demonstrate that these relationships were causal, we performed cheese-ripening tests by using bacterial isolates.

In the first part of our study, the multiomics approach could reveal correlations between the nonstarter bacteria and the volatile compounds, even though cheeses from the same categories were used (Japanese and French surface-mold ripened cheeses). The diversity and composition of bacterial microbiota in each surface-mold ripened cheese sample reflected the differences in the milk-processing methods, locations from where the samples were extracted, and the manufacturers. Cheese A was made from pasteurized cow’s milk and was dominated by LAB in both the core and rind. Cheese A was strongly influenced by the inoculated microorganisms, such as LAB and P. camemberti. The other cheeses, which were made from raw cow’s milk, had a more diverse bacterial microbiome than did cheese A. Generally, LAB dominates the core because of its anaerobic environment, whereas mold and yeast grow in the rind at the beginning of ripening due to its aerobic environment (3). These fungi deacidify the cheese surface, thereby promoting the development of various bacteria (3, 19–21). Moreover, the cheese microbiome is commonly formed by microorganisms derived from milk and cheese-making environments, as well as starter and secondary cultures (1, 3, 11, 22, 23). These facts are consistent with our results. On the other hand, the PCA for the volatile compounds indicated the differences in abundance of the ketones and the sulfur compounds among 42 surface-mold ripened cheese samples, but it did not clearly show the differences in milks (pasteurized or raw), cheese positions (core or rind), or manufacturers, as did the analysis of the bacterial microbiota. However, the Spearman's correlation analysis clearly showed correlations between the existence of the bacteria and the abundance of volatile compounds. In particular, the analysis showed correlations between nonstarter bacteria, such as LAB, *Actionbacteria*, and *Proteobacteria*, and ketones and sulfur compounds. Ketones and sulfur compounds are known to be the characteristic flavor compounds in surface mold-ripened cheeses (24, 25). The analysis suggested that differences in bacterial composition and their abundance ratios in cheeses, which are caused by differences in milk-processing methods and in cheese-making environments, contribute to the formation of cheese flavor. Thus, using a multiomics approach, we clearly demonstrated the statistical relationships between the bacteria and the flavors of cheeses of the same category.

In the second part of our study, we attempted to substantiate these correlations as causal relations. The results of the first part of this study and our previous research clearly revealed correlations between bacteria and components in soft-type ripened cheeses via the multiomics approach for the genus-level resolution analysis of the bacterial microbiota (7). These correlations did not rely on the differences in strain-level or cheese type. Therefore, the cheese-ripening tests were conducted using bacterial strains that satisfied the following two requirements: (i) the strains matched at the genus-level with the bacteria that correlated with the volatile compounds shown in the first part and (ii) the strains were isolated from sources that were different from the cheeses used for the first part.

The flavor-forming capabilities of *Carnobacterium* sp. and *Enterococcus* sp., which are known as NSLAB, have been reported in a few previous studies. Enterococci can degrade citrate into various aromatic compounds, such as acetate (26). Carnobacterium maltaromaticum isolated from milk produces 3-methylbutanal, 2-methylbutanal, and 2-methylpropanal (27, 28). The cheese-ripening test demonstrated that acetate increased in cheeses inoculated with *Enterococcus* sp. 8B5. In addition, 2-methylbutanal and 2-methylpropanal were detected in cheeses inoculated with *Carnobacterium* sp. SN-1-4. These results were consistent with those of previous studies. Moreover, cheeses inoculated with *Carnobacterium* sp. SN-1-4 contained ketones, such as 2-butanone, 2-pentanone, and 3-methyl-2-pentanone, which were relevant, as evidenced by the correlation analysis. The concentrations of propionic acid, butyric acid, 2-methylpropanoic acid, and 3-methlybutanoic acid, known as cheese flavor compounds (24, 25), increased in both of the cheeses that were inoculated with the two NSLAB strains. Thus, these results indicated that the NSLAB strains produced a variety of ketones and acids in addition to other, previously known components that are related to cheese flavor.

*Brevibacterium* spp. and *Micrococcuaceae* (including the genus *Glutamicibacter*) are known to produce sulfur compounds (19, 25, 29, 30). Deetae et al. (31) confirmed that Brevibacterium linens ATCC 9175 strain, two strains of *Glutamicibacter* (*Arthrobacter*) *arilaitensis* (Mu 107 and Po 102), and *Brachybacterium* sp. 111 strain, which were isolated from surface-ripened cheeses, produced sulfur compounds, such as dimethyl disulfide, and ketones, such as 2-butanone, on a casamino acid medium. In this study, a multiomics analysis revealed that *Actinobacteria* was correlated with ketones and that two genera of *Actinobacteria* (*Brevibacterium* and *Brachybacterium*) were correlated with sulfur compounds. Meanwhile, the cheese-ripening test revealed that the cheeses that were inoculated with three strains of *Actinobacteria* contained dimethyl disulfide after day 7 of ripening. Moreover, various sulfur compounds, such as dimethyl sulfide and methanthiol, and ketones, such as 2-butanone and 2,3-pentanedione, were detected in the cheeses that were inoculated with *Brevibacterium* sp. FU-2-6 and *Glutamicibacter* sp. FU-2-5, respectively. The results of the cheese-ripening tests are mostly consistent with those of the multiomics analysis and previous studies.

Research on the flavor-forming capability of *Proteobacteria* is limited to few strains. Irlinger et al. (32) reported that cheeses inoculated with Psychrobacter celer isolated from smear-ripened cheese contained volatile compounds such as aldehydes, ketones, and sulfur compounds after ripening. In addition, Deetae et al. (31, 33) confirmed that *Psychrobacter* spp. isolated from surface-ripened cheeses produced sulfur compounds and ketones. In our study, sulfur compounds, such as dimethyl disulfide and methanethiol, and ketones, such as 2-butanone and 2-pentanone, were detected in all of the cheeses that were inoculated with *Proteobacteria* during ripening. This finding is consistent with the results of the first part of our study and with the previous knowledge of *Psychrobacter*.

Overall, the results of the cheese-ripening test were largely in concordance with those of the multiomics analysis and with previous findings. Thus, we could substantiate the correlations revealed by the multiomics approach as causal relations, despite the validation of the bacteria having occurred at the genus-level. On the other hand, this result suggested the capability of nonstarter bacteria, such as *Proteobacteria*, to impart flavor to cheese, regardless of the bacterial isolation source. For cheese production, to screen for adjunct microbial cultures that improve the flavor of cheese, it is necessary to verify the capabilities of microorganisms at the strain-level. However, this process takes a long time and great effort, in that an infinite number of microorganisms must be considered. The present study demonstrates that the multiomics approach is an effective tool in such cases.

However, the results of the correlation analysis and cheese-ripening tests were not consistent for all compounds. For instance, although acids and esters were detected in cheeses inoculated with *Pseudoalteromonas* sp. TS-4-4, the relationship between *Pseudoalteromonas* sp. TS-4-4 and acids and esters was not found in the correlation analysis; similar findings were observed regarding the relationship of aldehydes and acids with *Carnobacterium* sp. SN-1-4, as well as regarding the relationship of acids with *Enterococcus* sp. 8B5 and *Psychrobacter* sp. FU-2-6. The viable counts of *Pseudoalteromonas* sp. TS-4-4 in the tested cheeses decreased from day 7 of ripening. Additionally, the composition of volatile compounds in the cheese on day 7 was distinct from those observed on days 14 and 21. Previous studies have indicated that the release of intracellular enzymes due to dead cells and autolysis may be responsible for flavor formation in cheese (34–36). Therefore, it is possible that the balance between intact and dead cells affected the composition of cheese compounds during ripening. Furthermore, the variety and abundance of volatile compounds vary, depending on the members of the cheese microbial community (37–39). Thus, the results of this study should be evaluated as the changes in cheese flavor components occur with the inoculation of a single strain. On the contrary, these findings provide an important insight into the potential of nonstarter bacteria and the mechanisms of flavor production in cheese communities where multiple microorganisms coexist.

In this study, we revealed the effectiveness of the multiomics approach and flavor-forming capability of nonstarter bacteria. At the same time, there is a need to examine the safety of these bacteria and their effect on human health with a focus on the evaluation of the possibility of their commercial use in cheese manufacturing. However, our results provide basic insights for understanding the role of nonstarter bacteria and for the development of future studies. Furthermore, our findings regarding the relationship between bacteria and volatile compounds can be applied for the quality control of cheese and can support the reproducible manufacturing of traditional fermented foods.

## MATERIALS AND METHODS

### Surface mold-ripened cheese samples.

Japanese and French surface mold-ripened soft cheese samples were obtained from a Japanese food market (Table 1). The Japanese samples included three cheese varieties from Factory A and two cheese varieties from Factory B. The French samples included one cheese variety per factory: Factory C and Factory D. For each cheese variety, three different lots were divided into cores and rinds. A total of 42 samples were used for each analysis. The NaCl concentration and pH were determined using a LAQUAtwin salt-22 salinity meter (HORIBA, Ltd., Kyoto, Japan) and a LAQUAtwin pH-11 pH meter (HORIBA, Ltd., Kyoto, Japan), respectively, as previously described (30).

### Sequencing of 16S rRNA gene amplicon.

The bacterial microbiota analysis was conducted as described by Unno et al. (7). The total DNA was extracted from 150 mg of cheese using a Quick-DNA Fecal/Soil Microbe Miniprep Kit (Zymo Research, Irvine, CA, United States). The bacterial amplicon libraries were prepared by amplifying the V3 and V4 regions of the 16S rRNA gene using a forward primer (5′-CCTACGGGNGGCWGCAG-3′) and a reverse primer (5′-GACTACHVGGGTATCTAATCC-3′) with an overhang adapter (40), following the Illumina protocol. After amplification, dual index and Illumina sequence adapters were added to the amplified products using a Nextera XT Index Kit (Illumina, San Diego, CA, USA). The polymerase chain reaction (PCR) products were purified with Agencourt AMPure XP (Beckman Coulter, Brea, CA, USA), and the read size was checked using an Agilent 2200 Tape Station (Agilent, Santa Clara, CA, USA). The libraries were diluted to 5 nM with 10 mM Tris (pH 8.5), based on the quantitative PCR (qPCR) results obtained using a Kapa Library Quantification Kit (Kapa Biosystems, Wilmington, MA, USA), and were sequenced on an Illumina MiSeq platform (Illumina, San Diego, CA, United States). The obtained reads were imported as paired-end sequence artifacts into the QIIME2 pipeline (41). The subsequent process followed the “Moving Pictures” tutorial in the QIIME2 user documentation (https://docs.qiime2.org/2019.4/tutorials/moving-pictures/). Quality filtering used DADA2 (42). Alpha and beta diversity were calculated in QIIME2. The alpha diversity analysis used the Shannon index (13), feature abundance (14), and Pielou’s evenness (15). The beta diversity analysis used the Jaccard similarity index (16). The bacteria were identified using a naive Bayes classifier that was pretrained on the Silva 16S rRNA gene database version 132 (43) in QIIME2.

### Isolation and identification of bacteria from ripened cheese.

Eight varieties of Japanese ripened soft cheese were used to isolate the bacterial strains. A rind sample of 0.5 g was homogenized in Ringer’s solution (per L [wt/vol]: 8.6 g NaCl, 0.3 g KCl, 0.43 g CaCl_2_·2H_2_O) using a BioMasher SP (Nippi, Tokyo, Japan). Aliquots of serially diluted suspensions were spread onto an isolation agar medium and were incubated aerobically at 15°C, 30°C, or 35°C for 3 days. The composition of the isolation medium is presented in Table S4. Selected representative isolates were restreaked on a new plate, based on colony morphology (color, shape, and size), and used for taxonomic assignment. Genomic DNA was extracted and purified using the DNeasy Blood and Tissue Kit (Qiagen, Hilden, Germany). The 16S rRNA gene was amplified using primers 27F (5′-AGAGTTTGATCMTGGCTCAG-3′) and 1492R (5′-TACGGYTACCTTGTTACGACTT-3′), as well as TaKaRa *Ex Taq* DNA polymerase (TaKaRa Bio, Inc., Shiga, Japan), according to the manufacturer’s instructions. The thermal cycling conditions were (i) 3 min at 95°C to initial denaturation; (ii) 30 cycles of 30 s at 95°C to denaturation, 15 s at 55°C to primer annealing, 1 min at 72°C to initial elongation; and (iii) 5 min at 72°C to final elongation. All of the amplified PCR fragments were purified via phenol-chloroform treatment and ethanol precipitation and were sent to Macrogen Japan Corp. (Tokyo, Japan) for sequencing. A similarity search of the isolates was performed using the NCBI BLAST tool (https://blast.ncbi.nlm.nih.gov/Blast.cgi) (44).

### Lab-scale cheese-ripening tests inoculated with nonstarter bacterial isolates.

Seven bacterial strains isolated from ripened soft cheese, Cobetia marina NBRC 102605 (provided by NBRC) (17), and one strain isolated from Brie cheese by Unno et al. (18) were used in the cheese-ripening tests (Table 2). All of the experiments were performed in triplicate. The bacteria were aerobically cultivated in 5 mL of liquid culture medium (Table 2) at 121 rpm at 30°C for 24 h. The cells were counted using a hemocytometer. These culture media were centrifuged for 5 min at 8,000 × *g* at 20°C, and the harvested cells were washed twice using Ringer’s solution. Thereafter, the washed cells were resuspended in Ringer’s solution to obtain cell suspensions of approximately 10^7^ cells/mL. These suspensions (100 μL) were inoculated into approximately 5 g core samples of commercially available, industrial, surface mold-ripened cheese (*n* = 3) that had been placed into 6-well plates (Greiner Bio-One, Kremsmünster, Austria) and heated at 55°C for 30 min. This industrial cheese was made from ultra-high-temperature sterilized milk and was pasteurized again after packaging. As a control, 100 μL of Ringer’s solution was added to cheese samples instead of the cell suspension. After inoculation, the plates were sealed with a sterile and breathable AeraSeal (Excel Scientific, Victorville, CA, United States) and incubated under aerobic conditions at 15°C with a relative humidity of approximately 80%. Cheese samples were collected on days 0, 7, 14, and 21 of ripening and were used to determine CFU and for the analysis of volatile compounds. The CFU were counted (CFU/g) using the culture medium supplemented with 1.5% agar (Table 2), as described in the previous section.

### Analysis of volatile compounds.

Surface mold-ripened cheese samples and cheese-ripening test samples were analyzed via HS-GC/MS, using the purge-and-trap technique. The volatile compounds analysis was performed using the GCMS-TQ8040 NX system with a HS-20 trap sampler (Shimadzu Corporation, Kyoto, Japan) that was equipped with an Inertcap WAX-HT column (0.25 mm × 60 m, df = 0.25 μm, GL Sciences, Inc., Tokyo, Japan), as described by Unno et al. (7). A sample of 0.1 g was placed in TORAST HS vials (Shimadzu GLC, Tokyo, Japan) and injected in split mode (5:1). The vials were agitated at 50°C for 30 min, and the temperature program was subsequently executed as follows: initially maintained at 50°C for 5 min, increased to 250°C at a rate of 10°C/min, and then kept at 250°C for 10 min. The carrier gas (helium) flow rate through the column was 1.7 mL/min. The mass spectrometry range was set between 33 to 400 *m/z*, and event time was 0.3 s in the scan mode. Peak identification was performed via a similarity search of the NIST17 mass spectral library (http://www.nist.gov) and a retention time comparison. The peaks detected between 1.5 to 35 min were retained for the analysis.

### Statistical analysis.

Statistical analyses and graphical outputs were performed using R packages (45). The heat maps and dendrograms were generated using the *gplots* package (46). PCA was performed using the *FactoMineR* (47) and *factoextra* packages (48). Correlation analysis was performed using the *psych* package (49), and the correlation coefficients were calculated using Spearman’s method. The correlation results were converted into a heat map using the *corrplot* package (50). The QIIME2 data were imported into *R* using the *qiime2R* package (51). The bar plot of alpha diversity metrices, the PCoA plot, and the time course changes in the volatile compounds during ripening were visualized using the *tidyverse* package (52). The statistical significance of the alpha and beta diversity values was determined via a Kruskal-Wallis test (53) and a PERMANOVA test (54) in QIIME2. Multiple comparisons were performed using Dunn’s test via the *dunn.test* package (55). The Benjamini-Hochberg false discovery rate (FDR) correction was used for the *P* value of the PERMANOVA test and Dunn’s test.

### Data availability.

Raw sequence data were deposited under the accession number DRA013549 in the DDBJ Sequence Read Archive. The partial sequence data of the 16S rRNA gene of the isolates in this study were deposited under accession numbers LC685115 to LC685121 in the DDBJ Sequence Read Archive.
