# Supplementary material for: Causality Verification for the Correlation between the Presence of Nonstarter Bacteria and Flavor Characteristics in Soft-Type Ripened Cheeses
Source: Microbiol Spectr. 2022 Nov 10;10(6):e02894-22. doi: 10.1128/spectrum.02894-22 (PMC9769828; doi:10.1128/spectrum.02894-22)
Supplement: Supplemental file 1 — Tables S1 to S6. Download spectrum.02894-22-s0001.pdf, PDF file, 0.3 MB [file spectrum.02894-22-s0001.pdf]

Table S1. Bacterial abundance data at the genus level generated by amplicon sequencing in Japanese and French surface mold-ripened cheeses used in this study.

Phylum	Genus	A1-core-1	A1-core-2	A1-core-3	A1-rind-1	A1-rind-2	A1-rind-3	A2-core-1	A2-core-2	A2-core-3	A2-rind-1	A2-rind-2	A2-rind-3	A3-core-1	A3-core-2	A3-core-3	A3-rind-1	A3-rind-2	A3-rind-3
Actinobacteria	Bifidobacterium	0	0	0	0	0	0	0	0	0	0	0	0	0	0	0	0	0	0
Actinobacteria	Corynebacterium 1	0	0	0	0	0	0	0	0	0	0	0	0	0	0	0	0	0	0
Actinobacteria	Actinomycetales bacterium JB111	0	0	0	0	0	0	0	0	0	0	0	0	0	0	0	0	0	0
Actinobacteria	Brevibacterium	0	0	0	0	2	0	0	0	0	0	0	0	0	0	0	0	0	0
Actinobacteria	Brachybacterium	0	0	0	0	0	0	0	0	0	0	0	0	0	0	0	0	0	0
Actinobacteria	Agrococcus	0	0	0	0	0	0	0	0	0	0	0	0	0	0	0	0	0	0
Actinobacteria	Leucobacter	0	0	0	0	0	0	0	0	0	0	0	0	0	0	0	0	0	0
Actinobacteria	Microbacterium	0	0	0	0	0	0	0	0	0	0	0	0	0	0	0	0	0	0
Actinobacteria	Parafrigoribacterium	0	0	0	0	0	0	0	0	0	0	0	0	0	0	0	0	0	0
Actinobacteria	D_4__Microbacteriaceae	0	0	0	0	0	0	0	0	0	0	0	0	0	0	0	0	0	0
Actinobacteria	Arthrobacter	0	0	0	0	0	0	0	0	0	0	0	0	0	0	0	0	0	0
Actinobacteria	Glutamicibacter	0	0	0	0	0	0	0	0	0	0	0	0	0	0	0	0	0	1
Actinobacteria	Yaniella	0	0	0	0	0	0	0	0	0	0	0	0	0	0	0	0	0	0
Actinobacteria	D_4__Micrococcaceae	0	0	0	0	0	0	0	0	0	0	0	0	0	0	0	0	0	0
Actinobacteria	Ruania	0	0	0	0	0	0	0	0	0	0	0	0	0	0	0	0	0	0
Actinobacteria	Streptomyces	0	0	0	0	0	0	0	0	0	0	0	0	0	0	0	0	0	0
Bacteroidetes	Flavobacterium	0	0	0	0	0	0	0	0	0	0	0	0	0	0	0	0	0	0
Bacteroidetes	Mesonia	0	0	0	0	0	0	0	0	0	0	0	0	0	0	0	0	0	0
Bacteroidetes	D_4__Flavobacteriaceae	0	0	0	0	0	0	0	0	0	0	0	0	0	0	0	0	0	0
Bacteroidetes	Chryseobacterium	0	0	0	0	0	0	0	0	0	0	0	0	0	0	0	0	0	0
Bacteroidetes	Pedobacter	0	0	0	0	0	0	0	0	0	0	0	0	0	0	0	0	0	0
Cyanobacteria	D_3__Chloroplast	0	0	0	0	0	0	0	0	0	0	0	0	0	0	0	0	0	0
Cyanobacteria	Arthrospira PCC-7345	0	0	1	0	0	0	0	0	0	0	0	0	0	0	0	0	0	0
Firmicutes	Geobacillus	0	0	0	0	0	0	0	0	0	0	0	0	0	0	0	0	0	0
Firmicutes	Jeotgalicoccus	0	0	0	0	0	0	0	0	0	0	0	0	0	0	0	0	0	0
Firmicutes	Staphylococcus	0	0	0	0	0	0	0	0	0	0	0	0	0	0	0	0	0	0
Firmicutes	Facklamia	0	0	0	0	0	0	0	0	0	0	0	0	0	0	0	0	0	0
Firmicutes	Alkalibacterium	0	0	0	0	0	0	0	0	0	0	0	0	0	0	0	0	0	0
Firmicutes	Carnobacterium	0	0	0	0	0	0	0	0	0	0	0	0	0	0	0	0	0	0
Firmicutes	Marinilactibacillus	0	0	0	0	0	0	0	0	0	0	0	0	0	0	0	0	0	0
Firmicutes	Enterococcus	0	0	0	0	0	0	0	0	0	0	3	0	0	0	0	0	0	0
Firmicutes	Vagococcus	0	0	0	0	0	0	0	0	0	0	0	0	0	0	0	0	0	0
Firmicutes	Lactobacillus	0	5	0	6	10	0	1	0	1	2	2	0	4	7	3	0	6	0
Firmicutes	Leuconostoc	3515	4689	5182	8467	6083	7860	6866	4987	7319	9913	7662	6876	5841	9772	8252	9478	11814	3920
Firmicutes	Weissella	0	0	0	0	0	0	0	0	0	0	0	0	0	0	0	0	0	0
Firmicutes	Lactococcus	36470	35278	34807	31506	33864	32122	33124	34987	32635	30058	32303	33091	34141	30196	31732	30504	28148	36065
Firmicutes	Streptococcus	14	19	2	19	28	2	7	26	45	10	27	27	14	14	8	17	18	0
Firmicutes	D_3__Lactobacillales	0	0	0	0	0	0	0	0	0	0	0	0	0	0	0	0	0	0
Firmicutes	Clostridiisalibacter	0	0	0	0	0	0	0	0	0	0	0	0	0	0	0	0	0	0
Firmicutes	Paeniclostridium	0	0	0	0	0	0	0	0	0	0	0	0	0	2	0	0	0	0
Firmicutes	Romboutsia	0	0	1	0	0	0	0	0	0	0	0	0	0	0	0	0	0	0
Proteobacteria	Albirhodobacter	0	0	0	0	0	0	0	0	0	0	0	0	0	0	0	0	0	0
Proteobacteria	D_4__Mitochondria	0	0	0	0	0	0	0	0	0	0	1	0	0	0	0	0	0	0
Proteobacteria	Idiomarina	0	0	0	0	0	0	0	0	0	0	0	0	0	0	0	0	0	0
Proteobacteria	Marinobacter	0	0	0	0	0	0	0	0	0	0	0	0	0	0	0	0	0	0
Proteobacteria	Pseudoalteromonas	1	0	4	0	0	0	0	0	0	0	0	0	0	0	0	0	0	0
Proteobacteria	Advenella	0	0	0	0	0	0	0	0	0	0	0	0	0	0	0	0	0	0
Proteobacteria	Delftia	0	0	0	0	2	0	0	0	0	0	0	0	0	0	0	0	0	1
Proteobacteria	D_4__Neisseriaceae	0	0	0	0	0	0	0	0	0	0	0	0	0	0	0	0	0	0
Proteobacteria	Citrobacter	0	0	0	0	0	0	0	0	0	0	0	0	0	0	0	0	0	0
Proteobacteria	Escherichia-Shigella	0	0	0	0	0	0	0	0	0	0	0	0	0	0	0	0	0	0
Proteobacteria	Hafnia-Obesumbacterium	0	0	0	0	0	0	0	0	0	0	0	0	0	0	2	0	0	0
Proteobacteria	Serratia	0	0	0	0	0	0	0	0	0	0	0	0	0	0	0	0	0	0
Proteobacteria	D_4__Enterobacteriaceae	0	0	0	0	0	0	0	0	0	0	0	0	0	0	0	0	0	0
Proteobacteria	Cobetia	0	0	0	0	0	0	0	0	0	0	0	0	0	0	0	0	0	0
Proteobacteria	Halomonas	0	0	0	0	0	0	0	0	0	0	0	0	0	0	0	0	0	0
Proteobacteria	Salinicola	0	0	0	0	0	0	0	0	0	0	0	0	0	0	0	0	0	0
Proteobacteria	Marinomonas	0	0	0	0	0	0	0	0	0	0	0	0	0	0	0	0	0	0
Proteobacteria	Acinetobacter	0	0	0	0	0	0	0	0	0	0	0	0	0	0	0	0	0	0
Proteobacteria	Psychrobacter	0	0	0	0	0	0	0	0	0	0	0	0	0	0	0	0	0	0
Proteobacteria	Pseudomonas	0	0	0	0	0	0	0	0	0	0	0	0	0	0	0	0	0	0
Other	Other	0	9	3	2	11	16	2	0	0	17	2	6	0	9	3	1	14	13

A subsampling of 40,00 reads per sample was performed without replacement.

B1-core-1	B1-core-2	B1-core-3	B1-rind-1	B1-rind-2	B1-rind-3	B2-core-1	B2-core-2	B2-core-3	B2-rind-1	B2-rind-2	B2-rind-3	C-core-1	C-core-2	C-core-3	C-rind-1	C-rind-2	C-rind-3	D-core-1	D-core-2	D-core-3	D-rind-1	D-rind-2	D-rind-3
0	0	0	0	0	0	0	0	0	0	0	0	0	0	2	1	0	0	0	0	0	0	0	0
1	0	0	5	0	0	0	0	0	0	0	0	0	0	0	0	0	9	0	0	0	14	0	0
0	0	0	0	0	0	0	0	0	0	0	0	0	0	0	0	0	1	0	0	0	0	0	0
66	0	0	3548	605	183	0	0	3	821	0	1352	0	1	6	5644	3051	427	84	0	28	4732	2103	98
23	0	0	37	1	4	0	0	0	1654	3	43	0	0	0	419	1647	388	0	0	12	1077	608	11
0	0	0	0	0	0	0	0	0	0	0	0	0	0	0	0	174	0	0	0	0	0	0	0
0	0	0	0	0	0	0	0	0	0	0	0	0	0	0	5	668	57	0	0	0	0	0	0
0	0	0	0	0	0	0	0	0	0	0	0	0	0	0	0	84	0	0	0	0	0	0	0
0	0	0	0	3	0	0	0	0	0	0	0	0	0	0	0	0	0	0	0	0	0	0	0
0	0	0	0	0	0	0	0	0	0	0	0	0	0	0	0	2	0	0	0	0	0	0	0
0	0	0	0	0	0	0	0	0	34	0	0	0	0	0	0	0	0	0	0	0	0	0	0
0	0	0	0	0	0	0	0	0	9	0	0	61	0	55	2840	10159	862	0	0	22	497	875	64
0	0	0	0	0	0	0	0	0	0	0	0	0	0	0	0	0	2	0	0	0	0	0	0
0	0	0	0	0	0	0	0	0	0	0	0	0	0	0	39	60	78	30	2	26	455	2807	20
0	0	0	0	0	0	0	0	0	0	0	0	0	0	0	0	0	5	0	0	0	0	0	0
0	0	0	0	0	0	0	0	0	0	0	0	0	0	0	0	0	17	0	0	0	0	0	0
0	0	0	0	0	0	0	0	0	0	0	0	0	0	0	0	0	0	0	0	0	3	0	0
0	0	0	0	0	0	0	0	0	0	0	0	0	0	0	12	0	0	0	0	0	0	0	0
0	0	0	0	0	0	0	0	35	0	0	21	0	0	0	0	0	0	0	0	0	0	0	0
0	0	0	0	0	0	0	0	0	0	0	0	0	0	0	0	0	0	0	0	4	0	0	6
0	0	0	0	0	0	0	0	0	0	0	0	0	0	0	0	0	0	0	0	0	0	6	0
0	0	0	0	4	12	0	0	0	0	0	0	0	0	0	0	0	0	0	0	0	0	0	0
0	0	0	0	0	0	0	0	0	0	0	0	0	0	0	0	0	0	0	0	0	0	0	0
0	0	0	0	0	0	0	0	0	0	0	0	0	0	0	0	0	0	0	0	0	0	14	17
0	0	0	0	0	0	0	0	0	0	0	0	3	0	0	0	22	6	0	0	0	14	145	40
20	6	3	3	0	7	39	10	10	1	3	2	33	0	11	698	844	235	643	6	18	2614	1981	242
1	0	0	0	0	0	0	0	0	0	0	0	0	0	0	0	0	0	0	0	0	0	0	0
0	0	0	0	0	0	0	0	0	0	0	0	0	0	0	0	0	0	0	0	0	8	0	0
0	0	0	0	0	0	0	0	0	0	0	0	83	5	0	8145	22	127	0	0	0	46	0	0
0	0	0	0	23	2	0	0	0	0	0	0	0	0	0	0	0	0	0	0	0	0	0	0
0	0	0	0	0	0	0	0	0	0	0	0	105	166	1	2339	1938	3700	0	0	0	0	0	0
0	0	0	0	0	0	0	0	0	0	0	0	0	0	0	0	110	48	0	0	0	0	0	0
1212	3508	4153	2190	2525	3636	2144	4457	3102	2200	3249	3046	3685	5153	6396	1922	863	3263	3175	1128	1564	8552	2206	2935
35	3	18	65	8	20	15	7	2	36	13	25	787	1478	733	2519	2033	2661	26	0	0	106	6	3
0	0	0	0	0	0	0	0	0	0	0	0	0	0	0	11	0	0	0	0	0	0	0	0
19048	21418	20168	6422	17129	19422	20521	22969	20374	6393	23461	15645	34020	30453	32739	11279	11745	26271	35937	38773	38143	3859	22767	18374
11407	14970	12147	8213	12747	12687	14565	12371	12009	12382	13062	8671	51	0	7	32	354	61	0	0	0	0	7	0
0	0	0	0	0	0	0	0	0	0	0	0	0	2	0	0	0	0	0	0	0	0	0	0
0	0	2	0	0	0	0	0	0	0	0	0	0	0	0	0	0	0	0	0	0	0	0	0
0	0	0	0	0	0	0	0	0	0	0	0	0	0	1	0	0	0	0	0	0	0	0	2
0	0	0	0	0	0	0	0	0	0	0	0	0	0	0	0	0	0	0	0	0	0	0	0
0	0	0	0	0	12	0	0	0	0	0	0	0	0	0	0	0	0	0	0	0	0	0	0
0	0	0	0	0	0	0	0	0	0	0	0	0	0	0	0	0	0	0	0	0	0	0	0
0	0	0	0	0	0	0	0	0	0	0	22	0	0	0	0	0	0	0	0	0	0	0	0
0	0	0	0	0	0	0	0	0	0	0	0	0	0	0	0	0	0	0	0	0	12035	8	7
6024	0	226	7066	2215	1269	27	0	206	2881	160	2507	0	0	0	0	0	0	0	0	62	1721	3816	18044
0	0	0	0	0	0	0	0	0	0	0	0	0	0	0	0	0	3	0	0	0	0	0	0
0	0	0	0	0	0	0	0	0	0	0	0	0	0	0	0	0	0	0	0	0	0	0	0
0	0	0	0	0	0	0	0	0	0	0	0	0	0	0	0	0	0	0	0	0	2	0	0
0	0	0	0	0	0	0	0	0	0	0	0	0	0	0	0	78	0	0	0	0	0	0	0
0	0	0	0	0	0	0	0	0	0	0	0	26	0	0	0	0	0	0	0	0	0	0	0
1812	90	3225	10229	1300	1461	2606	181	4188	3309	25	7289	1103	2712	35	1779	2243	1314	28	89	99	9	21	15
12	0	0	0	0	0	0	0	0	0	0	2	0	0	0	0	0	0	0	0	0	0	0	0
0	0	0	0	0	0	0	0	0	0	0	0	27	0	0	4	25	0	0	0	0	0	0	0
2	0	55	753	3268	1230	0	0	0	1972	22	1106	0	0	0	0	0	0	0	0	0	0	7	0
24	0	2	133	15	42	3	0	0	346	0	81	0	0	2	28	1990	3	3	0	7	84	27	40
0	0	0	0	0	0	0	0	0	1	0	0	0	0	0	0	0	0	0	0	0	0	0	0
3	0	0	916	6	0	0	0	71	0	0	172	0	0	0	0	0	0	0	0	0	0	0	0
0	0	0	0	0	0	0	0	0	0	0	0	5	2	4	0	0	0	0	1	12	0	3	14
310	0	0	418	140	8	80	0	0	7959	0	16	9	28	6	2251	1884	457	51	0	3	4147	2543	54
0	0	0	0	0	0	0	0	0	0	0	0	0	0	0	0	0	0	0	0	0	0	9	4
0	5	1	2	11	5	0	5	0	2	2	0	2	0	2	33	4	5	23	1	0	25	41	10

Table S2. Peak areas of volatile compounds detected in Japanese and French surface mold-ripened cheeses used in this study.

Sample	Acetic acid	Butanoic acid	3-Methylbutanoic acid	Hexanoic acid	Isopropyl Alcohol	Ethanol	1-Nonanol	2-Butanol	2-Methyl-1-propanol	2-Pentanol	1-Methoxy-2-propanol	1-Butanol	Isopentyl alcohol
A1-core-1	0	0	0	0	122752	2676293	0	0	140386	0	0	0	650400
A1-core-2	0	0	0	0	242310	1595765	0	0	78463	0	0	0	1107446
A1-core-3	0	0	0	0	0	869173	0	0	97676	0	0	0	1197326
A1-rind-1	0	0	0	0	2752573	3335869	0	0	391470	0	0	0	1535132
A1-rind-2	0	0	0	0	675943	24770610	0	0	819419	0	0	0	3788844
A1-rind-3	0	0	0	0	412120	13402439	0	0	1435928	0	0	0	3050340
A2-core-1	0	0	0	0	264060	732094	0	0	0	130248	0	0	51482
A2-core-2	0	0	0	0	0	1597425	0	0	87173	0	0	0	847360
A2-core-3	0	0	0	0	0	2657618	0	0	252159	0	0	0	3085409
A2-rind-1	0	615239	0	700708	2348799	33650943	0	0	1122665	334792	0	0	3471119
A2-rind-2	0	0	0	0	1202556	58931860	0	0	1728514	189254	0	0	9298118
A2-rind-3	0	0	0	0	822619	16144805	0	0	1800398	0	0	0	2289051
A3-core-1	0	0	0	0	1638466	26903193	0	0	1413447	0	0	0	3176784
A3-core-2	0	0	0	0	835399	5053110	0	0	517167	0	0	0	5777727
A3-core-3	0	0	0	0	300858	1520221	0	0	96468	0	0	0	1396095
A3-rind-1	0	0	0	0	1646392	25925391	0	0	1990087	220729	0	0	4764519
A3-rind-2	0	0	0	0	1395807	23452480	0	0	1267319	0	0	0	6386651
A3-rind-3	0	0	0	0	0	10506044	0	588270	1259045	0	0	0	1288644
B1-core-1	0	0	0	0	1221548	0	0	0	0	0	0	0	417857
B1-core-2	0	0	0	0	334912	1322645	0	0	0	0	0	0	0
B1-core-3	409275	655716	0	0	0	689067	0	0	0	0	0	0	671185
B1-rind-1	0	0	0	0	586106	650805	0	0	0	0	0	0	835429
B1-rind-2	0	0	0	0	523219	5712048	0	0	0	0	0	0	0
B1-rind-3	0	0	0	0	724351	0	871183	0	0	552843	0	0	0
B2-core-1	0	0	0	0	1489952	158068	0	0	0	90325	0	0	990027
B2-core-2	0	0	0	0	284912	628054	0	0	0	0	0	0	0
B2-core-3	348910	776359	0	305957	266867	1180314	0	0	0	0	0	0	591281
B2-rind-1	0	0	0	0	8912300	3496415	0	606381	0	304976	0	0	1398965
B2-rind-2	0	0	0	0	683932	5993481	0	0	154464	1651875	0	0	862186
B2-rind-3	0	0	0	0	1952677	3005030	0	0	74315	105677	0	0	360233
C-core-1	728188	0	399239	0	3577995	204225	0	735677	0	573311	0	0	260022
C-core-2	0	0	0	0	343245	26121798	0	282627	0	0	0	0	0
C-core-3	0	0	0	0	0	28983229	0	0	0	108849	50074	39148	489119
C-rind-1	834441	0	0	0	1034675	0	0	0	0	98040	0	0	1069887
C-rind-2	0	0	0	0	1158538	834420	0	0	0	0	0	0	0
C-rind-3	0	0	0	0	4461248	650222	531915	462194	0	1105269	0	0	233593
D-core-1	0	0	0	0	7953056	3876178	0	293581	0	3919991	0	76610	1588256
D-core-2	0	171719	0	0	0	16955931	0	0	0	0	0	0	0
D-core-3	0	0	0	0	0	5231686	0	0	0	0	0	0	49132
D-rind-1	691943	0	0	0	508665	0	0	0	0	91745	0	0	278259
D-rind-2	0	0	0	0	2001236	1332554	0	0	0	0	0	0	0
D-rind-3	0	0	0	0	1127686	161362	131940	0	0	106337	0	0	0

2-Hexanol	2-Heptanol	1-Octen-3-ol	2-Ethylhexanol	2-Nonanol	2-Methylbutanal	3-Methylbutanal	Ethyl Acetate	Propyl acetate	Ethyl butanoate	Isopropyl butyrate	Butyl acetate	Pentyl acetate	Ethyl hexanoate	Hexyl acetate
0	0	0	0	0	0	140942	0	0	0	0	0	0	0	0
0	0	0	0	0	0	1081885	0	0	0	0	0	0	0	0
0	0	0	0	0	0	2700100	0	0	0	0	0	0	0	0
0	0	0	0	0	0	0	0	0	0	0	0	0	0	0
0	334556	0	0	0	0	314216	0	277237	68446	0	0	246549	0	0
0	0	0	0	0	0	530108	0	85495	69463	0	0	248255	0	0
0	150431	0	0	0	0	0	0	0	0	0	0	0	0	0
0	0	0	0	0	0	789683	0	0	0	0	0	81384	0	0
0	0	0	0	0	0	4656119	0	0	0	0	0	0	0	0
0	575590	0	0	508997	0	0	0	156567	142673	0	0	325177	62226	0
0	997564	0	0	629192	0	517834	0	1164376	251936	0	40313	1061669	0	70145
0	0	0	0	0	0	0	0	0	0	0	0	0	0	0
0	0	0	0	0	135797	473946	0	0	0	0	0	0	0	0
0	0	0	0	0	0	4331228	0	0	0	0	0	0	0	0
0	0	0	0	0	0	4046661	0	0	0	0	0	0	0	0
0	428493	0	0	0	0	234581	0	71694	0	0	0	0	0	0
0	0	0	0	0	0	759937	0	0	0	0	0	0	0	0
0	0	0	0	0	0	0	0	0	0	0	0	0	0	0
0	0	0	0	0	0	1489212	0	0	0	0	0	0	0	0
0	0	0	0	0	0	0	342542	0	0	0	0	0	0	0
0	0	0	0	0	0	1732718	0	0	0	0	0	0	0	0
0	0	0	0	0	0	172759	0	0	0	0	0	0	0	0
0	0	0	0	0	0	0	0	0	0	0	0	0	0	0
0	696311	0	0	0	0	0	0	332716	0	0	0	107244	0	0
0	0	0	0	0	211436	1474887	0	0	0	0	0	0	0	0
0	0	0	0	0	0	0	139139	0	0	0	0	0	0	0
0	0	0	0	0	0	364925	312234	0	0	0	0	0	0	0
0	0	0	0	0	109465	512852	172955	0	343547	604318	0	0	0	0
106463	2107112	0	0	454094	0	0	0	405263	0	0	0	495834	0	0
0	286338	119715	0	0	0	0	0	207020	0	0	0	41295	0	0
0	0	0	0	0	0	630845	0	0	0	0	0	0	0	0
0	0	0	0	0	0	0	72831	0	0	0	0	0	0	0
0	0	0	0	0	0	0	71897	0	0	0	0	0	133956	0
0	0	0	0	0	0	364160	0	0	0	0	0	0	0	0
0	0	0	0	0	0	0	0	0	0	0	0	0	0	0
134655	460983	0	564048	1120511	0	0	0	193561	0	0	0	0	0	0
153955	1796992	0	0	0	0	1537888	0	0	0	0	0	0	0	0
0	0	0	0	0	0	0	50622	0	0	0	0	0	0	0
0	0	0	0	0	0	104957	23683	0	0	0	0	0	0	0
0	0	0	0	0	0	1063045	0	0	0	0	0	0	0	0
0	0	0	0	0	0	0	61436	0	0	0	0	0	0	0
0	291454	0	0	0	0	0	0	0	0	0	0	0	0	0

Heptyl acetate	6-Heptenyl acetate	Nonyl acetate	Acetone	2-Butanone	2-Pentanone	Diacetyl	3-Methyl-2-pentanone	2-Hexanone	5-Hexen-2-one	2-Heptanone	3-Octanone	2-Octanone	Acetoin	2-Nonanone	8-Nonen-2-one	2-Decanone	2-Undecanone	Methanethiol
0	0	0	2381370	0	78423	0	0	0	0	235964	0	0	0	268082	0	0	0	0
0	0	0	2525580	0	0	588963	0	0	0	64182	0	0	3146406	86304	0	0	0	0
0	0	0	5432635	38050	0	405839	0	0	0	0	0	0	1230962	83621	0	0	0	0
0	0	0	21548333	85949	0	0	0	0	0	67765	0	0	64616	0	0	0	0	126212
1200191	0	0	6001453	109376	2826225	0	0	75566	0	2192294	0	56763	400364	2087269	131265	0	0	0
0	0	415826	3906755	91990	435055	0	0	42602	0	374196	0	0	0	0	0	1341566	0	0
0	0	0	662531	0	2779669	0	0	112777	0	3020699	0	65945	60499	1548134	153606	0	0	0
0	0	0	3081212	0	0	0	0	0	0	1386430	0	0	6663303	1288741	0	0	0	0
0	0	0	5420407	0	0	0	0	0	0	0	0	0	1639555	0	0	0	0	0
345719	0	0	12282208	87965	2764589	0	0	174685	0	3710583	0	168044	0	7866219	521451	0	818233	0
2676409	125272	0	8145528	286422	7987038	0	0	300552	0	6583431	0	228202	463397	9593765	0	0	1913091	0
0	0	0	3571841	54039	0	0	0	0	0	0	0	0	0	0	0	0	0	0
0	0	0	13098827	0	0	0	0	0	0	0	0	0	84284	0	0	0	0	0
0	0	0	9914771	45618	0	233558	0	0	0	97419	0	0	1354931	0	0	0	0	0
0	0	0	7783410	99701	0	148930	0	0	0	0	0	0	653607	0	0	0	0	0
243259	0	0	11550030	112775	2938061	0	0	242332	0	2980120	0	87404	70340	2983812	0	0	0	0
0	0	0	12681264	0	350682	0	0	0	0	229301	0	0	1962338	162615	0	0	0	0
0	0	383250	2094528	54408	0	0	0	0	0	1491624	0	0	0	0	0	1172416	0	0
0	0	0	13810135	1112032	1337810	0	0	0	0	145735	0	0	0	0	0	0	0	466282
0	0	0	435536	0	0	1923404	0	0	0	0	0	0	5408767	0	0	0	0	0
0	0	0	1561424	0	15084709	0	0	63160	0	2815275	0	0	49201698	709974	137672	0	0	0
0	0	0	11462796	908570	1538285	0	0	0	0	329982	0	0	0	131954	0	0	0	1529353
0	0	0	3334939	0	1806559	0	0	0	0	0	0	0	0	4221529	0	0	0	0
436805	0	0	13574113	821362	19724330	0	0	751482	0	14614987	170515	340379	0	23455191	3021505	0	2398297	0
0	0	0	17673740	1262474	2639939	0	0	0	0	643318	0	0	414976	251619	0	0	0	569214
0	0	0	836442	0	3113894	1095629	0	0	0	0	0	0	3351125	2968586	0	0	0	0
0	0	0	872525	62078	0	7772997	0	0	0	171929	0	0	18665609	71865	0	0	0	0
0	0	0	37039055	2735383	4416396	0	0	0	0	779552	0	0	286093	553120	0	0	0	2370164
932810	0	0	5740302	385475	27843506	0	0	804202	164545	22661808	0	366002	150197	17440769	1571827	0	797584	0
175393	0	0	21597386	352132	5258007	0	0	0	0	2001272	401469	38602	0	3133693	317783	0	0	0
0	0	0	11358785	1962802	4075703	0	0	0	0	1221755	0	0	0	0	0	0	0	0
0	0	0	315794	232136	0	0	0	0	0	0	0	0	0	0	0	0	0	0
0	0	0	635615	212180	1040151	0	0	41707	0	266055	0	0	120151	0	0	0	0	0
0	0	0	24686080	5215815	6288626	0	932065	0	0	1695481	0	0	0	5398782	0	0	0	990775
0	0	0	12049285	365811	735677	0	0	0	0	1667238	0	0	0	3068808	0	0	0	0
219575	0	0	38867277	3185205	25610177	0	0	1118696	0	5901087	0	395294	55813	18546870	903989	0	3826039	0
0	0	0	18822045	707370	14544251	0	0	597015	49916	8104450	0	92191	3894255	596697	0	0	0	0
0	0	0	2751796	217380	0	1636161	0	0	0	1446390	0	0	0	824174	0	0	0	0
0	0	0	2213572	171173	0	715134	0	0	0	242937	0	0	256634	101762	0	0	0	0
0	0	0	28376589	3696052	7929458	0	780276	0	0	619316	0	0	522858	0	0	0	419751	0
0	0	687790	17423298	238120	0	0	0	0	0	0	0	0	0	0	0	1365020	0	0
68282	0	0	27793596	635436	2370028	0	0	133746	0	4682997	23770	183161	35182	9553881	462016	0	0	0

Dimethyl sulfide	S-Methyl thioacetate	Dimethyl disulfide	S-Methyl 3-methylbutanethioate	2,4-Dithiapentane	Dimethyl trisulfide	1-Heptene	1,3-Octadiene	2,2,4,6,6-Pentamethylheptane	Toluene	Styrene	2,6-Dimethylpytazine
0	0	106848	0	0	0	0	0	0	0	0	0
0	0	0	0	0	0	0	0	0	0	0	0
0	0	0	0	0	0	0	0	31370	0	0	0
680523	274096	5013533	0	0	336981	0	0	0	0	0	0
0	0	0	0	0	0	0	0	0	57505	0	0
815094	0	187560	0	0	0	0	0	55577	82229	0	0
62603	0	208249	0	0	0	0	0	0	0	0	0
0	0	0	0	0	0	0	0	0	0	0	0
0	0	99942	0	0	0	0	0	24625	0	0	0
89616	0	82601	0	0	0	411540	0	0	0	172867	0
0	0	0	0	0	0	0	0	0	67223	0	0
501057	0	0	0	0	0	0	0	0	0	0	0
205528	0	0	0	0	0	0	0	0	0	0	0
0	0	0	0	0	0	0	0	0	0	0	0
0	0	0	0	0	0	0	0	34427	0	0	0
262907	0	108965	0	0	94811	0	0	0	0	0	0
0	0	0	0	0	0	0	0	39325	0	0	0
142403	0	0	0	0	0	0	0	0	0	0	0
13404273	127812	39557094	0	716324	779796	0	0	0	0	0	0
1117263	0	2074189	0	0	0	0	0	0	0	0	0
229209	0	227475	0	0	0	0	0	0	122267	0	0
6412635	776603	37658995	138813	827068	5915291	0	0	0	0	0	0
3672301	164205	7899379	0	0	0	0	0	0	0	0	0
1340908	0	312500	0	0	0	807883	0	0	136127	243527	0
10044884	232688	43665836	0	213108	1017372	0	0	0	0	0	0
263520	0	294940	0	0	0	0	0	0	0	0	0
5932745	172378	2726484	0	0	0	0	0	0	184148	0	0
9224858	2705670	48103065	117019	367737	3382378	0	0	0	0	0	123919
397209	0	1543593	0	0	0	90878	0	0	147695	764207	0
17583506	221065	11380944	0	336178	316695	0	92069	0	119652	0	0
866235	0	4433678	0	0	515555	0	0	0	0	0	0
692744	0	6399119	0	0	0	0	0	0	0	0	0
397080	0	375395	0	0	0	0	0	0	25066	0	0
963750	0	23966189	0	0	1057335	0	0	0	0	0	0
385177	0	5652811	0	0	511981	0	0	0	0	0	0
372195	0	164182	0	0	0	122131	0	0	0	0	0
256473	0	212533	0	0	172303	0	0	0	0	0	0
35010	0	0	0	0	0	0	0	0	0	0	0
284081	0	0	0	44385	0	0	0	0	26534	0	0
212427	0	11880858	0	0	0	0	0	0	0	0	0
51118	0	0	0	0	0	0	0	0	0	0	0
853946	0	579345	0	0	151827	0	0	0	61213	120095	0

Table S3. The loadings for the components PC1–PC5 of the principal component analysis performed on the surface-mold ripened cheeses.

Compounds	Category	PC1	PC2	PC3	PC4	PC5
Acetic acid	Acid	-0.124	0.092	-0.157	-0.094	0.015
Butanoic acid	Acid	0.007	-0.141	-0.042	0.270	0.704
3-Methylbutanoic acid	Acid	-0.058	0.062	-0.074	-0.177	-0.076
Hexanoic acid	Acid	0.117	-0.084	0.061	0.152	0.499
Isopropyl Alcohol	Alcohol	0.187	0.714	0.052	-0.324	0.053
Ethanol	Alcohol	0.308	-0.324	0.697	-0.079	0.070
1-Nonanol	Alcohol	0.569	0.170	-0.379	0.118	-0.194
2-Butanol	Alcohol	0.056	0.488	-0.035	-0.390	-0.031
2-Methyl-1-propanol	Alcohol	0.158	-0.332	0.537	-0.123	-0.112
2-Pentanol	Alcohol	0.525	0.235	-0.300	-0.356	0.151
1-Methoxy-2-propanol	Alcohol	-0.054	-0.103	-0.008	-0.072	0.179
1-Butanol	Alcohol	0.137	0.055	-0.175	-0.358	0.201
Isopentyl alcohol	Alcohol	0.250	-0.239	0.713	-0.144	-0.077
2-Hexanol	Alcohol	0.609	0.224	-0.377	-0.383	0.054
2-Heptanol	Alcohol	0.818	0.069	-0.068	-0.081	0.184
1-Octen-3-ol	Alcohol	0.000	0.115	-0.050	0.719	-0.357
2-Ethylhexanol	Alcohol	0.415	0.215	-0.259	-0.260	-0.259
2-Nonanol	Alcohol	0.752	0.094	0.114	-0.168	-0.059
2-Methylbutanal	Aldehyde	-0.158	0.411	0.172	-0.023	0.010
3-Methylbutanal	Aldehyde	-0.191	-0.104	0.023	-0.162	-0.074
Ethyl Acetate	Ester	-0.208	0.122	0.023	0.154	0.589
Propyl acetate	Ester	0.759	-0.097	0.578	0.183	-0.084
Ethyl butanoate	Ester	0.210	0.462	0.734	-0.039	0.215
Isopropyl butyrate	Ester	-0.127	0.777	0.295	-0.077	0.238
Butyl acetate	Ester	0.453	-0.153	0.785	0.019	-0.086
Pentyl acetate	Ester	0.671	-0.172	0.643	0.088	0.069
Ethyl hexanoate	Ester	0.019	-0.119	0.031	-0.046	0.286
Hexyl acetate	Ester	0.453	-0.153	0.785	0.019	-0.086
Heptyl acetate	Ester	0.680	-0.139	0.635	0.100	-0.020
6-Heptenyl acetate	Ester	0.453	-0.153	0.785	0.019	-0.086
Nonyl acetate	Ester	-0.113	-0.140	0.028	-0.123	-0.141
Acetone	Ketone	0.207	0.691	-0.067	-0.118	-0.351
2-Butanone	Ketone	0.095	0.598	-0.126	-0.228	-0.265
2-Pentanone	Ketone	0.824	0.277	-0.339	-0.031	0.037
Diacetyl	Ketone	-0.135	-0.131	-0.076	0.270	0.607
3-Methyl-2-pentanone	Ketone	-0.080	0.131	-0.096	-0.129	-0.225
2-Hexanone	Ketone	0.892	0.176	-0.311	-0.154	-0.017
5-Hexen-2-one	Ketone	0.537	0.068	-0.242	-0.066	0.280
2-Heptanone	Ketone	0.865	0.100	-0.231	0.078	0.149
3-Octanone	Ketone	0.161	0.133	-0.158	0.784	-0.358
2-Octanone	Ketone	0.948	0.113	-0.144	0.028	-0.026
Acetoin	Ketone	-0.072	-0.132	-0.117	0.170	0.492
2-Nonanone	Ketone	0.876	0.134	-0.217	0.145	-0.075
8-Nonen-2-one	Ketone	0.713	0.122	-0.393	0.285	0.008
2-Decanone	Ketone	-0.111	-0.155	0.047	-0.127	-0.145
2-Undecanone	Ketone	0.808	0.142	-0.051	-0.047	-0.191
Methanethiol	Sulfur compound	-0.228	0.872	0.265	-0.009	0.095
Dimethyl sulfide	Sulfur compound	-0.181	0.544	0.058	0.637	-0.123
S-Methyl thioacetate	Sulfur compound	-0.188	0.857	0.302	0.036	0.215
Dimethyl disulfide	Sulfur compound	-0.277	0.784	0.171	0.141	-0.059
S-Methyl 3-methylbutanethioate	Sulfur compound	-0.188	0.748	0.260	0.036	0.142
2,4-Dithiapentane	Sulfur compound	-0.219	0.599	0.146	0.338	-0.113
Dimethyl trisulfide	Sulfur compound	-0.223	0.725	0.200	0.072	0.029
1-Heptene	Other	0.539	0.065	-0.254	0.250	0.067
1,3-Octadiene	Other	0.000	0.115	-0.050	0.719	-0.357
2,2,4,6,6-Pentamethylheptane	Other	-0.141	-0.230	0.087	-0.118	-0.148
Toluene	Other	0.443	-0.083	-0.100	0.613	0.366
Styrene	Other	0.633	0.042	-0.257	0.141	0.271
2,6-Dimethylpytazine	Other	-0.127	0.777	0.295	-0.077	0.238

Volatile compounds data of Japanese and French surface-mold ripened cheeses are used for principal component analysis.

Table S4. Composition of the medium used in this study

Medium	PCAM (+ NaCl)	PCAM + V + CV (+NaCl)	Funke (+ NaCl)	MB (+ Glucose)	Artifical Seawater medium	B1P5 (+ NaCl)	TSB (+ NaCl)
Composition	Plate Count Agar 23.5 g	Plate Count Agar 23.5 g	Peptone 10 g	Marine Broth 37.4 g	Yeast Extract 20 g	Tryptone 10 g	Tryptic Soy Broth 30 g
	Skim milk 10 g	Skim milk 10 g	Yeast extract 5 g	Glucose 0 or 2 g	Casein, from milk 30 g	Fish extract 50 g	Yeast extract 2.5 g
	NaCl 0 or 30 g	NaCl 0 or 30 g	Malt extract 5 g	Agar 15 g	Agar 15 g	NaCl 0 or 25 or 50 g	NaCl 0 or 30 g
		Vancomycin ※5 mg	Casamino acid 5 g			KCl 1 g	
		Crystal violet ※5 mg	Tween 80 10 g			$MgSO_4 \cdot 7H_2O$ 2.5 g	
			NaCl 0 or 30 g				
			Agar 15 g				
	Distilled water 1000 ml	Distilled water 1000 ml	Distilled water 1000 ml	Distilled water 1000 ml	Distilled artifical seawater 1000 ml	Distilled water 1000 ml	Distilled water 1000 ml

Autoclave at 121º C.

※ - Dissolved in distilled water and sterilized by filtration through a membrane with a 0.2 µm pore size. This solution was added to medium after autoclave.

Table S5. Peak areas of volatile compounds detected in cheeses inoculated with bacterial isolates.

Sample	Acetic acid	Propanoic acid	Butanoic acid	2-Methylpropanoic acid	3-Methylbutanoic acid	2-Methylbutanoic acid	Hexanoic acid	Octanoic acid	Ethanol	2-Methyl-1-propanol	Isopentyl alcohol	Isopropyl Alcohol	1-Butanol	2-Heptanol	1-Methoxy-2-propanol
Control-0-1	136164	0	0	0	0	0	0	0	7002428	124597	245091	288559	0	0	0
Control-0-2	0	0	0	0	0	0	0	0	34839441	397831	1149325	0	76946	0	29297
Control-0-3	0	0	0	0	0	0	0	0	30865355	208972	1148078	0	78928	0	78505
Control-7-1	0	0	0	0	0	0	0	0	28417740	0	173278	174838	0	0	0
Control-7-2	0	0	0	0	0	0	0	0	29676626	0	195225	329807	0	0	0
Control-7-3	0	0	0	0	0	0	0	0	25754877	0	142372	205075	0	0	0
Control-14-1	0	0	0	0	0	0	0	0	33496709	0	84193	365720	0	0	0
Control-14-2	0	0	0	0	0	0	0	0	33965762	0	76139	516407	0	0	0
Control-14-3	0	0	0	0	0	0	0	0	28067350	0	61942	389186	0	0	0
Control-21-1	337313	0	0	0	0	0	0	0	21221864	0	0	0	0	0	0
Control-21-2	0	0	0	0	0	0	0	0	35389329	0	0	195518	0	0	0
Control-21-3	0	0	0	0	0	0	0	0	27774313	0	0	303368	0	0	0
Car-0-1	0	0	0	0	0	0	0	0	47671053	187410	584388	0	0	0	0
Car-0-2	0	0	0	0	0	0	0	0	45526839	217511	832332	0	92682	0	79133
Car-0-3	0	0	0	0	0	0	0	0	59537034	236846	777517	0	0	0	0
Car-7-1	0	0	144308	0	402261	0	0	0	117269	0	52105	0	0	0	0
Car-7-2	0	0	0	0	1036341	0	0	0	74619	0	121453	0	0	0	0
Car-7-3	0	0	108821	0	2279652	0	0	0	83708	0	61589	0	0	0	0
Car-14-1	0	0	172585	0	1364074	0	0	0	246624	0	0	0	0	0	0
Car-14-2	0	0	284333	0	2440558	0	0	0	315141	0	0	0	0	0	0
Car-14-3	0	0	545083	92908	4209644	0	0	0	626126	0	0	0	0	0	0
Car-21-1	0	0	1577567	151437	3558881	0	0	0	320066	0	0	0	0	0	0
Car-21-2	0	0	686770	155625	2158046	0	0	0	505730	0	0	0	0	0	0
Car-21-3	0	84328	1392326	213006	4939336	0	0	0	435241	0	0	0	0	0	0
Ent-0-1	252085	0	0	0	0	0	0	0	2722467	694871	0	0	0	0	0
Ent-0-2	790125	0	0	0	0	0	0	0	1915640	644787	0	0	105995	0	0
Ent-0-3	988709	0	0	0	0	0	0	0	2879411	187221	553254	0	234218	0	0
Ent-7-1	1631483	0	0	0	0	0	0	0	2149215	0	0	0	0	0	174354
Ent-7-2	1344150	0	0	0	0	0	0	0	2377119	0	0	0	0	0	131730
Ent-7-3	992968	0	0	0	0	0	0	0	2220178	0	0	0	0	0	206605
Ent-14-1	7615752	0	768388	724763	3228556	0	0	0	2109932	0	0	0	0	0	233515
Ent-14-2	40400762	0	5004026	4805022	21984431	0	0	0	2372063	0	0	0	0	0	371513
Ent-14-3	7040154	0	599200	548627	2968998	0	0	0	2180313	0	0	0	0	0	0
Ent-21-1	98488325	206398	5043253	11449647	49595553	0	0	0	2210503	0	0	0	0	0	0
Ent-21-2	42689823	0	1663484	4123819	19599918	0	0	0	3188353	0	0	0	0	0	0
Ent-21-3	102323239	224570	4671822	10893132	48714992	0	0	0	1454083	0	0	0	0	0	0
Bra-0-1	0	0	0	0	0	0	0	0	5362932	0	52220	151926	0	0	0
Bra-0-2	0	0	0	0	0	0	0	0	6612821	0	21964	157793	0	0	0
Bra-0-3	0	0	0	0	0	0	0	0	4716945	0	44621	154029	0	0	0
Bra-7-1	0	0	0	0	0	0	0	0	1560677	0	0	0	0	0	0
Bra-7-2	0	0	0	0	0	0	0	0	1050363	0	0	0	0	0	0
Bra-7-3	0	0	0	0	0	0	0	0	1637142	0	0	0	0	0	0
Bra-14-1	0	0	0	0	0	0	0	0	9340494	0	0	0	0	0	0
Bra-14-2	0	0	0	0	0	0	0	0	9195586	0	0	0	0	0	0
Bra-14-3	0	0	0	0	0	0	0	0	3734546	0	0	0	0	0	0
Bra-21-1	0	0	0	254282	0	951875	0	0	1022186	0	0	0	0	0	0
Bra-21-2	0	0	0	0	0	0	0	0	3105535	0	0	0	0	0	0
Bra-21-3	0	0	0	0	0	96891	0	0	1762049	0	0	0	0	0	0
Bre-0-1	0	0	0	0	0	0	0	0	6224494	42369	149002	150666	0	29747	0
Bre-0-2	0	0	0	0	0	0	0	0	5654869	43739	140109	160279	0	23284	0
Bre-0-3	0	0	0	0	0	0	0	0	4217552	77194	198055	155868	0	40511	0
Bre-7-1	0	0	0	0	0	0	0	0	0	0	0	0	0	0	0
Bre-7-2	0	0	0	0	0	0	0	0	0	0	0	0	0	0	0
Bre-7-3	0	0	0	0	0	0	0	0	0	0	0	0	0	0	46095
Bre-14-1	0	0	0	0	0	0	0	0	0	0	0	0	0	0	0
Bre-14-2	0	0	0	0	0	0	0	0	0	0	0	0	0	0	0
Bre-14-3	0	0	0	0	0	0	0	0	0	0	0	0	0	0	0
Bre-21-1	0	0	0	0	0	0	0	0	0	0	0	0	0	0	0
Bre-21-2	445489	0	394433	82633	0	0	0	0	0	0	0	0	0	0	0
Bre-21-3	1142389	0	1933048	114608	0	0	1404891	471261	0	0	0	0	0	0	0

Gul-0-1	0	0	0	0	0	0	0	0	5910696	92531	193405	0	0	0	0
Gul-0-2	0	0	0	0	0	0	0	0	5914608	127656	212606	0	0	0	0
Gul-0-3	0	0	0	0	0	0	0	0	5798840	148205	200882	0	0	0	0
Gul-7-1	0	0	0	0	0	0	0	0	0	0	0	0	0	0	0
Gul-7-2	0	0	0	0	0	0	0	0	0	0	23493	0	0	0	0
Gul-7-3	0	0	0	0	0	0	0	0	0	0	0	0	0	0	0
Gul-14-1	0	0	0	0	0	0	0	0	0	0	0	0	0	0	0
Gul-14-2	0	0	0	0	0	0	0	0	0	0	0	0	0	0	0
Gul-14-3	0	0	0	0	0	0	0	0	0	0	0	0	0	0	0
Gul-21-1	0	0	0	0	0	0	0	0	0	0	0	0	0	0	0
Gul-21-2	0	0	0	0	0	0	0	0	0	0	0	0	0	0	0
Gul-21-3	0	0	0	0	0	0	0	0	0	0	0	0	0	0	0
Cob-0-1	0	0	0	0	0	0	0	0	6877274	115439	163736	172341	0	0	0
Cob-0-2	0	0	0	0	0	0	0	0	5607615	113879	153161	198668	0	0	0
Cob-0-3	0	0	0	0	0	0	0	0	5558839	118841	135020	134646	0	0	0
Cob-7-1	0	0	0	0	0	0	0	0	0	0	0	0	0	0	0
Cob-7-2	0	0	0	0	0	0	0	0	0	0	0	0	0	0	0
Cob-7-3	0	0	0	0	0	0	0	0	0	0	0	0	0	0	0
Cob-14-1	0	0	0	0	0	0	0	0	0	0	0	0	0	0	0
Cob-14-2	0	0	0	0	0	0	0	0	0	0	0	0	0	0	0
Cob-14-3	0	0	0	0	0	0	0	0	0	0	0	0	0	0	0
Cob-21-1	0	0	0	0	0	0	0	0	0	0	0	0	0	0	0
Cob-21-2	0	0	0	0	0	0	0	0	0	0	0	0	0	0	0
Cob-21-3	0	0	0	0	0	0	0	0	0	0	0	0	0	0	0
Hal-0-1	0	0	0	0	0	0	0	0	4056053	0	78333	214413	0	0	0
Hal-0-2	0	0	0	0	0	0	0	0	5377650	46806	75218	0	0	0	0
Hal-0-3	0	0	0	0	0	0	0	0	5413156	0	83166	0	0	0	0
Hal-7-1	0	0	0	0	0	0	0	0	941986	0	0	0	0	0	0
Hal-7-2	0	0	0	0	0	0	0	0	1175400	0	0	0	0	0	0
Hal-7-3	0	0	0	0	0	0	0	0	1158905	0	0	0	0	0	0
Hal-14-1	0	0	0	0	0	0	0	0	1543603	0	0	0	0	0	0
Hal-14-2	0	0	0	0	0	0	0	0	2115564	0	0	0	0	0	0
Hal-14-3	0	0	0	0	0	0	0	0	2968058	0	0	0	0	0	0
Hal-21-1	0	0	0	0	0	0	0	0	2508997	0	0	0	0	0	0
Hal-21-2	0	0	0	0	0	0	0	0	1302700	0	0	0	0	0	0
Hal-21-3	0	0	0	0	0	0	0	0	2388634	0	0	0	0	0	0
Pse-0-1	0	0	0	0	0	0	0	0	4120705	108546	122148	0	0	0	0
Pse-0-2	0	0	0	0	0	0	0	0	2521075	92103	154424	0	0	0	0
Pse-0-3	0	0	0	0	0	0	0	0	3609146	91238	115216	0	0	0	0
Pse-7-1	679762	0	816962	890690	3555291	0	0	0	0	0	162393	42296	0	0	0
Pse-7-2	0	0	755174	770792	3133687	0	254249	0	0	0	0	0	0	0	0
Pse-7-3	983058	78426	2769602	2481604	9944926	0	457701	0	428616	0	0	0	0	0	0
Pse-14-1	81396	0	1448871	926031	6156396	0	722670	0	1640085	0	0	0	0	0	0
Pse-14-2	464187	146282	3603010	1983417	13455445	0	1729617	471173	1446144	0	0	0	0	0	0
Pse-14-3	104939	0	1246095	745973	5089568	0	626467	0	3498329	0	0	0	0	0	0
Pse-21-1	510425	188420	6694222	2775860	0	20533825	4196655	0	1589675	0	0	0	0	0	0
Pse-21-2	601796	326498	7364596	5264999	38582027	0	6351015	1094243	1998780	0	0	0	0	0	0
Pse-21-3	664317	214539	5657985	3817440	0	24621941	3343044	732163	2825209	0	0	0	0	0	0
Psy-0-1	0	0	0	0	0	0	0	0	29391754	88964	515385	256896	0	0	0
Psy-0-2	0	0	0	0	0	0	0	0	47780608	301232	4452467	0	0	0	0
Psy-0-3	0	0	0	0	0	0	0	0	46519379	206773	2564520	0	0	0	0
Psy-7-1	1093475	0	485447	108376	1165902	0	0	0	0	0	1396286	0	0	0	0
Psy-7-2	1714551	0	512386	41282	780151	0	0	0	0	0	1101170	0	0	0	0
Psy-7-3	120044	0	0	0	281987	0	0	0	0	0	768198	0	0	0	0
Psy-14-1	0	0	0	0	359857	0	0	0	0	0	613997	0	0	0	0
Psy-14-2	63944	0	0	0	136138	0	0	0	0	0	221342	0	0	0	0
Psy-14-3	163545	0	122754	154829	1303551	0	0	0	0	0	637818	0	0	0	0
Psy-21-1	0	0	0	0	428465	0	0	0	0	0	66452	0	0	0	0
Psy-21-2	725417	0	292184	402102	2064875	0	0	0	0	0	122061	0	0	0	0
Psy-21-3	940553	0	524057	793904	1731889	0	246276	0	0	0	69217	0	0	0	0

Car, *Carnobacterium* sp. SN-1-4; Ent, *Enterococcus sp.* 8B5; Bra, *Brachybacterium* sp. FU-4-2; Bre, *Brevibacterium* sp. FU-2-6, Gul, *Gultamicibacter* sp. FU-2-5; Cob, *Cobetia marina* NBRC102605; Hal, *Halomonas* sp. SN-2-8; Pse, *Pseudoalteromonas* sp. TS-4-4; Psy, *Psychrobacter* sp. FU-2-4

The sample name separated by a hyphen indicates the control or bacterial name, ripening days, and sample number from the front.

3-Methylbutanal	Acetaldehyde	2-Methylpropanal	2-Methylbutanal	Ethyl Acetate	2-Hydroxyethyl propanoate	Ethyl propanoate	Ethyl butanoate	Ethyl hexanoate	Acetone	2-Heptanone	Diacetyl	Acetoin	2-Butanone	2-Pentanone	2-Nonanone	2,3-Pentanedione
0	0	0	0	4626802	0	0	0	0	1840783	130372	176186	48096	0	0	0	0
0	0	0	0	4088293	0	0	0	0	1155459	0	677864	0	0	0	0	0
0	0	0	0	3840432	0	0	0	0	1097393	251092	592480	474162	0	0	0	0
0	0	0	0	642566	577125	0	0	0	209019	472875	752618	2610930	0	0	0	0
59023	0	0	0	698656	684531	0	0	0	275341	300408	615483	1870690	0	0	0	0
39057	0	0	0	568446	729420	0	0	0	168073	193889	440013	1773055	0	0	0	0
51340	0	0	0	1073530	1012005	0	0	0	188086	137216	486727	1683173	0	0	0	0
53412	0	0	0	1435642	1502709	0	0	0	193580	90452	488036	1314300	0	0	0	0
46123	0	0	0	971928	1032852	0	0	0	144973	157374	275436	1252144	0	0	0	0
0	509165	0	0	0	186269	0	0	0	236008	0	0	0	0	0	0	0
0	0	0	0	584933	834744	0	0	0	195407	53014	529058	2253500	0	0	0	0
60734	0	0	0	638755	387534	0	0	0	243252	149805	528394	2637652	0	0	0	0
286414	0	0	0	2067954	118126	0	0	0	707756	724007	397704	1638473	0	0	0	0
0	0	0	0	3051687	0	0	0	0	721204	412422	891540	0	0	0	0	0
0	0	0	0	3544082	0	0	0	0	862338	825224	635013	618494	0	0	0	0
766287	0	87186	0	0	0	0	0	0	0	55059	0	0	0	81305	0	0
597500	0	103730	100438	0	0	0	0	0	0	33599	0	0	13208	30055	0	0
306663	0	38227	0	0	0	0	0	0	0	0	0	0	0	0	0	0
282231	0	79172	93939	0	0	0	0	0	65442	0	0	0	27870	156199	0	0
336670	0	127194	134457	0	0	0	0	0	0	0	0	0	24606	111640	0	0
669838	0	276890	284120	0	0	0	0	0	0	0	0	0	70718	241147	0	0
250025	0	0	155573	0	0	0	0	0	233236	38541	0	0	0	233883	0	0
405689	0	256777	279854	0	0	0	0	0	0	0	0	0	59950	296405	0	0
190554	0	0	158230	0	0	0	0	0	411093	0	0	0	58577	311027	0	0
0	0	0	0	769097	0	0	0	0	677308	0	521249	0	69969	333122	0	0
0	0	0	0	979906	0	0	0	0	676623	0	584854	0	71985	298231	0	0
0	0	0	0	775129	0	0	0	0	795491	402495	415047	1435971	94996	258494	0	0
91075	0	0	0	672569	0	0	0	0	116709	265397	1766032	20504602	0	0	0	0
251456	0	0	0	780193	0	0	0	0	155338	232103	1940385	22656647	0	0	0	0
118003	0	0	0	560770	0	0	0	0	82330	128409	1617986	23251960	0	0	0	0
533786	0	0	0	697142	0	0	0	0	51905	223688	1626905	17527904	0	0	0	0
1018345	0	0	0	748277	0	0	0	0	229788	106572	1325030	8385258	0	0	0	0
186328	0	0	0	641194	0	0	0	0	80123	261452	1419849	14808534	0	0	0	0
370479	0	0	0	671876	0	0	0	0	188617	0	1113743	4779631	0	0	0	0
470361	0	0	0	546180	0	0	0	0	224949	268649	1558460	8385775	0	0	0	0
912632	0	0	0	767282	0	0	0	0	283905	0	1133649	0	0	0	0	0
0	0	0	0	3848909	51072	0	0	0	1282037	48577	331851	558502	0	0	0	0
0	0	0	0	5406522	33919	0	0	0	1061323	37330	351553	620262	0	0	0	0
0	0	0	0	6612338	21574	0	0	0	926601	59927	526532	737957	0	0	0	0
0	0	0	0	57596	0	0	0	0	41162	0	383295	3673645	0	0	0	0
0	0	0	0	50441	27718	0	0	0	58646	0	362905	4374015	0	0	0	0
0	0	0	0	51492	0	0	0	0	69348	0	421145	1860803	0	0	0	0
0	0	0	0	51939	150087	0	0	0	52828	0	311449	3183921	0	0	0	0
0	0	0	0	79885	103944	0	0	0	52212	0	367528	4119222	0	0	0	0
0	0	0	0	39227	0	0	0	0	49845	0	284835	4577216	0	0	0	0
0	0	0	0	109009	0	0	0	0	55363	0	202520	4787926	0	0	0	0
0	0	0	0	122744	0	0	0	0	43413	0	233885	2397062	0	0	0	0
0	0	0	0	166820	0	0	0	0	43358	0	315829	2583728	0	0	0	0
0	0	0	0	4482473	50348	0	0	0	1052720	115022	204223	342870	0	0	0	0
0	0	0	0	3085866	32147	0	0	0	1145345	58118	119333	254393	0	0	0	0
0	0	0	0	3378808	24782	0	0	0	1339464	91753	147386	287333	0	0	0	0
0	0	0	0	0	0	0	0	0	4389552	0	0	0	0	0	0	0
0	0	0	0	0	0	0	0	0	3401658	0	309806	0	273200	718601	0	0
0	0	0	0	0	0	0	0	0	5700870	79323	177041	97828	316041	1356294	0	0
0	0	0	0	0	0	0	0	0	586184	22975	0	0	0	70982	0	0
0	0	0	0	0	0	0	0	0	611191	0	0	0	0	0	0	0
0	0	0	0	0	0	0	0	0	735305	0	0	0	0	0	0	0
0	0	0	0	0	0	0	0	0	527666	0	0	0	0	92400	0	0
0	0	0	0	0	0	0	0	0	2351637	0	0	0	0	0	0	0
0	0	0	0	0	0	0	0	0	877020	0	0	0	0	0	0	0

25040	0	0	0	4589445	0	0	0	0	1038832	99969	116649	367211	0	0	0	0	
48162	0	0	0	4039929	27751	0	0	0	1990614	114410	117184	309181	0	0	0	0	
0	0	0	0	2761654	0	0	0	0	1733039	97622	142956	314567	0	0	0	0	
0	0	0	0	120231	87311	0	0	0	1171367	30411	981369	131697	663156	0	0	25009	
0	0	0	0	108482	0	0	0	0	632053	32286	948132	94380	166080	0	0	38987	
0	0	0	0	101984	0	0	0	0	626050	29097	422634	70412	181271	0	0	0	
0	0	0	0	53708	0	0	0	0	1331178	16409	709829	101153	484729	0	0	27477	
0	0	0	0	62642	0	0	0	0	1044566	0	594986	88477	419594	0	0	22030	
0	0	0	0	41801	0	0	0	0	1319129	47968	537053	135713	871438	0	0	0	
0	0	0	0	82715	0	0	0	0	2687196	0	518605	360944	1849349	0	0	0	
0	0	0	0	70733	0	0	0	0	993287	0	295151	238513	789661	0	0	0	
0	0	0	0	94828	0	0	0	0	1209077	0	512888	308133	834495	0	0	0	
0	0	0	0	3188859	69880	0	0	0	1330461	176993	147236	378598	0	0	0	0	
0	0	0	0	2811100	52790	0	0	0	1185600	100262	84517	354688	0	0	0	0	
0	0	0	0	3197146	28537	0	0	0	1812906	127076	106405	321877	0	0	0	0	
0	0	0	0	54619	0	0	0	0	812638	26369	0	0	62908	124177	0	0	
0	0	0	0	41034	0	0	0	0	1124575	22577	0	0	91500	143708	0	0	
0	0	0	0	51585	0	0	0	0	710296	0	0	0	75585	147795	0	0	
0	0	0	0	29671	0	0	0	0	720113	0	0	0	202470	289021	0	0	
0	0	0	0	36580	0	0	0	0	432560	0	0	0	137132	314228	0	0	
0	0	0	0	46746	0	0	0	0	403552	0	0	0	109773	228423	0	0	
0	0	0	0	54541	0	0	0	0	182026	24778	0	0	126337	297532	0	0	
0	0	0	0	44073	0	0	0	0	397458	0	0	0	239134	352493	0	0	
0	0	0	0	19593	0	0	0	0	142788	0	0	0	153989	106378	0	0	
0	0	0	0	6956224	34624	0	0	0	769682	107679	828651	1940207	0	0	85810	0	
0	0	0	0	5698927	106507	0	0	0	562445	87453	819026	1649182	0	0	0	0	
0	0	0	0	5065278	57216	0	0	0	876256	71778	879710	2447348	0	0	0	0	
0	0	0	0	52196	0	0	0	0	135745	0	23139	0	0	21990	0	0	
0	0	0	0	54102	0	0	0	0	99322	0	0	0	0	71994	0	0	
0	0	0	0	47563	0	0	0	0	91634	0	0	0	0	48451	0	0	
0	0	0	0	36029	0	0	0	0	105349	0	0	0	231140	135434	0	0	
0	0	0	0	67010	0	0	0	0	215534	0	0	0	129062	146154	0	0	
0	0	0	0	49476	40809	0	0	0	134062	0	0	0	48933	71542	0	0	
0	0	0	0	121643	0	0	0	0	54631	0	35168	159629	0	0	0	0	
0	0	0	0	0	0	0	0	0	25154	0	0	0	0	0	0	0	
0	0	0	0	148279	0	0	0	0	28206	0	27021	39417	0	0	0	0	
25490	0	0	0	2144502	0	0	0	0	484643	117684	147502	434158	0	0	0	0	
14951	0	0	0	2866266	0	0	0	0	612242	134450	137943	487539	0	0	0	0	
20910	0	0	0	2845209	0	0	0	0	1727000	197224	169664	327441	0	0	0	0	
232896	0	0	14384	59753	0	0	0	0	2930006	37729	0	257564	180431	695598	0	0	
398144	0	0	0	54404	0	0	0	0	2406194	46795	0	225614	169671	732063	0	0	
166904	0	0	0	64254	0	0	910568	0	1576734	45976	0	260306	101961	922441	0	26469	
113067	0	0	0	36597	75191	0	1481146	133257	35244	0	109329	86282	0	0	0	58048	
323988	0	0	0	45434	0	0	3047160	253277	92016	85025	280388	0	0	0	0	105686	
0	0	0	0	0	0	0	0	0	72647	0	191720	0	0	0	0	0	
160718	0	0	0	141986	0	0	2456563	302913	0	0	120442	0	0	0	0	71881	
320120	0	0	0	162141	0	0	4373713	262340	0	0	223877	84573	0	0	0	73424	
253289	0	198097	56283	199368	0	0	3173446	153511	0	0	272279	510874	0	0	0	42432	
118038	0	0	0	1059448	0	0	0	0	405404	253375	288591	173848	0	0	0	0	
6273525	0	0	0	2148351	0	195758	0	0	2051622	736312	1088542	2223646	0	0	0	292880	
2052665	0	0	0	1988812	84598	61623	0	0	1183372	595733	865847	1959545	0	0	0	133052	
469082	0	0	0	34095	0	0	0	0	1330254	41424	0	0	367294	600314	0	0	
328907	0	0	0	22787	0	0	0	0	606409	0	0	0	347412	434632	0	0	
165308	0	0	0	24057	0	0	0	0	493739	42640	0	0	284050	346913	0	0	
171641	0	0	0	33886	0	0	0	0	558016	24208	0	0	775560	397460	0	0	
55492	0	0	0	37709	0	0	0	0	1281452	37071	0	49178	679300	505629	0	0	
86842	0	0	0	42412	0	0	0	0	1321145	0	0	0	828604	495283	0	0	
32510	0	0	0	172615	0	0	0	0	1396004	80765	0	45297	514440	335144	0	0	
55946	0	0	0	98570	0	0	0	0	1721748	24879	0	32675	535237	288485	0	0	
75128	0	0	0	112035	0	0	0	0	1576307	22997	0	83179	400460	252614	0	0	

3-Methyl-2-pentanone	Methyl Isobutyl Ketone	3-Hydroxy-3-methyl-2-butanone	Dimethyl sulfide	Dimethyl disulfide	Dimethyl trisulfide	Methanethiol	*S*-Methyl thioacetate	1,2-Dimethoxyethane	Chloroform	Cyclopentanone	Pyridine	Toluene	2,5-Dimethylpyrazine
0	0	0	0	0	0	0	0	0	0	0	0	0	0
0	0	0	0	0	0	0	0	1017525	0	0	0	0	0
0	0	0	0	0	0	0	0	829349	0	0	0	0	0
0	0	0	0	0	0	0	0	0	37443	0	130032	0	0
0	0	0	0	0	0	0	0	0	39103	0	129351	0	0
0	0	0	0	0	0	0	0	0	25317	0	121652	0	0
0	0	0	0	0	0	0	0	0	57881	0	0	0	0
0	0	0	0	0	0	0	0	0	75378	0	0	0	0
0	0	0	0	0	0	0	0	0	26860	0	0	0	0
0	0	0	0	0	0	0	0	0	0	0	0	0	0
0	0	0	0	0	0	0	0	0	48327	0	216029	40647	0
0	0	0	0	0	0	0	0	0	40215	0	221958	0	0
0	0	0	0	0	0	0	0	725309	0	0	0	0	0
0	0	0	0	0	0	0	0	966822	0	0	0	0	0
0	0	0	0	0	0	0	0	815598	0	0	0	0	0
86757	0	0	0	0	0	0	0	0	0	0	0	0	0
0	0	0	0	0	0	0	0	0	0	0	0	0	0
0	0	0	0	0	0	0	0	0	0	0	0	0	0
72932	0	0	0	0	0	0	0	0	0	0	0	0	0
85798	0	0	0	0	0	0	0	0	0	0	0	0	0
135567	0	0	0	0	0	0	0	0	0	0	0	0	0
98687	0	0	0	0	0	0	0	0	0	0	0	0	0
131912	0	0	0	0	0	0	0	0	0	0	0	0	0
84628	0	0	0	0	0	0	0	0	0	0	0	0	0
0	0	0	0	0	0	0	0	784730	0	0	0	0	0
0	0	0	0	0	0	0	0	895589	0	0	0	0	0
0	0	0	0	0	0	0	0	741603	0	0	0	0	0
0	0	0	0	0	0	0	0	0	0	0	0	0	0
0	0	0	0	0	0	0	0	0	0	0	0	0	0
0	0	0	0	0	0	0	0	0	0	0	0	0	0
0	0	0	0	0	0	0	0	0	0	0	0	0	0
0	0	0	0	0	0	0	0	0	0	0	45305	0	0
0	0	0	0	0	0	0	0	0	0	0	0	0	0
0	0	0	0	0	0	0	0	0	0	0	0	0	0
0	0	0	0	0	0	0	0	0	0	0	0	0	0
0	0	0	0	0	0	0	0	0	0	0	0	0	0
0	0	0	0	0	0	0	0	0	0	0	0	0	0
0	0	0	0	0	0	0	0	0	0	0	0	0	0
0	0	0	0	0	0	0	0	0	0	0	0	0	0
26267	0	0	0	23587	0	0	0	0	0	0	0	0	0
43732	0	0	0	19479	0	0	0	0	0	0	0	0	0
0	0	0	0	0	0	0	0	0	0	0	0	0	0
0	0	0	0	0	0	0	0	0	0	0	0	0	0
0	0	0	0	58586	0	0	0	0	0	0	0	0	0
38237	0	0	0	83603	0	0	0	0	0	0	0	0	0
0	0	0	0	32204	0	0	0	0	0	0	0	0	0
0	0	0	0	30927	0	0	0	0	0	0	0	0	0
0	0	0	0	34250	0	0	0	0	0	0	0	0	0
0	0	0	0	0	0	0	0	0	0	0	0	0	0
0	0	0	0	0	0	0	0	0	0	0	0	0	0
0	0	0	0	0	0	0	0	0	0	0	0	0	0
0	0	0	0	0	0	0	0	0	0	0	0	0	0
0	0	0	0	610415	0	0	0	0	47543	0	0	26650	0
0	0	0	0	1229047	60827	63074	0	0	49226	44235	90691	0	0
0	0	0	0	323519	0	41602	0	0	0	0	0	0	0
0	0	0	49847	0	0	98541	0	0	0	0	0	0	0
0	0	0	113593	0	0	140966	0	0	0	0	0	0	0
0	0	0	0	219809	0	0	0	0	0	0	0	0	0
0	0	0	130860	0	0	177511	0	0	0	0	0	0	0
0	0	0	0	0	0	0	0	0	0	0	0	0	0

0	0	0	0	0	0	0	0	160204	0	0	0	0	0
0	0	0	0	0	0	0	0	179440	0	0	0	0	0
0	0	0	0	0	0	0	0	131740	0	0	0	0	0
27196	20114	0	0	223903	0	0	0	0	0	0	0	0	0
0	0	0	0	25332	0	0	0	0	0	0	0	0	0
0	0	0	0	59312	0	0	0	0	0	0	0	0	0
0	0	0	0	79461	0	0	0	0	0	0	0	0	0
0	14061	0	0	167626	0	0	0	0	0	0	0	0	0
0	0	0	0	296757	0	0	0	0	0	0	0	0	0
0	0	0	0	267252	0	0	0	0	0	0	0	0	0
0	0	0	0	388832	0	0	0	0	0	0	0	0	0
0	0	0	0	105595	0	0	0	0	0	0	0	0	0
0	0	0	0	0	0	0	0	0	0	0	0	0	0
0	0	0	0	0	0	0	0	0	0	0	0	0	0
0	0	0	0	0	0	0	0	0	0	0	0	0	0
0	0	0	0	90121	0	0	0	0	0	0	0	0	0
39414	0	0	0	150265	0	0	0	0	0	0	0	0	0
33056	0	0	0	75995	0	0	0	0	0	0	0	0	0
50646	0	0	0	486124	0	49491	0	0	0	0	0	0	0
0	0	0	0	391017	0	36185	0	0	0	0	0	0	0
0	0	0	0	397868	0	31005	0	0	0	0	0	0	0
0	0	0	0	576686	0	80670	32456	0	0	0	0	0	0
95132	0	0	35056	716835	0	144587	0	0	0	0	0	0	0
0	0	0	0	771398	0	165901	21343	0	0	0	0	0	0
0	0	0	0	0	0	0	0	0	0	0	0	0	0
0	0	0	0	0	0	0	0	170561	0	0	0	0	0
0	0	0	0	0	0	0	0	170692	0	0	0	0	0
0	0	0	0	46627	0	0	0	0	0	0	0	0	0
0	0	0	0	145934	0	0	0	0	0	0	0	0	0
0	0	0	0	93113	0	0	0	0	0	0	0	0	0
0	0	0	0	437473	0	35266	0	0	0	0	0	0	0
0	0	0	0	345600	0	22117	20012	0	0	0	0	0	0
0	0	0	0	261961	0	35601	0	0	0	0	0	0	0
0	0	0	0	22041	0	0	0	0	0	0	0	0	0
0	0	0	0	0	0	0	0	0	0	0	0	0	0
0	0	0	0	46119	0	0	0	0	0	0	0	0	0
0	0	0	0	0	0	0	0	154146	0	0	0	0	0
0	0	0	0	0	0	0	0	141077	0	0	0	0	0
0	0	0	0	0	0	0	0	133447	0	0	0	0	0
0	0	0	884465	4246013	0	251002	181855	0	0	0	0	0	0
0	0	0	1003365	5440956	48058	300514	190447	0	0	0	0	0	0
0	0	0	309611	3135468	78547	240890	52832	0	0	0	0	0	0
0	0	48404	0	437006	0	41142	0	0	0	0	0	0	36813
0	0	0	0	588272	35604	0	0	0	0	0	0	0	389744
0	0	0	0	523243	0	0	0	0	0	0	0	0	0
0	0	0	0	243794	0	0	0	0	0	0	0	0	0
0	0	0	0	200986	0	0	0	0	0	0	0	0	0
0	0	0	0	282179	0	0	0	0	0	0	0	0	0
0	0	0	0	0	0	0	0	0	0	0	0	0	0
0	0	0	0	0	0	0	0	0	0	0	0	0	0
0	0	0	0	0	0	0	0	470075	0	0	0	0	0
0	0	0	0	516612	0	0	0	0	0	0	0	0	0
44108	0	0	0	689038	0	43681	0	0	0	0	0	0	0
0	0	0	0	625933	0	65350	22549	0	0	0	0	0	0
121446	0	0	64155	1235217	44401	33055	30551	0	0	0	0	0	0
101290	0	0	0	601127	0	22564	0	0	0	0	0	0	0
0	0	0	0	563977	0	0	0	0	0	0	0	0	0
43489	0	0	0	547048	0	36488	0	0	0	0	0	0	0
32252	0	0	0	455372	0	37523	0	0	0	0	0	0	0
0	0	0	0	395252	0	44602	0	0	0	0	0	0	0

Table S6. The loadings for the components PC1–PC5 of the principal component analysis performed on the cheese-ripening test samples.

Compounds	Category	PC1	PC2	PC3	PC4	PC5
Acetic acid	Acid	0.815	0.076	0.000	0.133	-0.050
Propanoic acid	Acid	0.811	-0.486	0.067	0.208	0.124
Butanoic acid	Acid	0.872	-0.398	0.054	0.149	0.048
2-Methylpropanoic acid	Acid	0.901	-0.299	0.039	0.207	0.081
3-Methylbutanoic acid	Acid	0.650	-0.205	0.028	-0.077	0.546
2-Methylbutanoic acid	Acid	0.478	-0.370	0.050	0.365	-0.560
Hexanoic acid	Acid	0.776	-0.505	0.071	0.133	0.101
Octanoic acid	Acid	0.685	-0.487	0.065	0.244	0.142
Ethanol	Alcohol	-0.794	-0.244	-0.084	0.349	0.186
2-Methyl-1-propanol	Alcohol	-0.346	0.050	0.875	0.075	0.035
Isopentyl alcohol	Alcohol	-0.416	0.057	0.832	0.282	0.158
Isopropyl Alcohol	Alcohol	-0.567	-0.165	-0.538	0.211	0.104
1-Butanol	Alcohol	-0.308	0.018	0.848	0.290	0.189
1-Methoxy-2-propanol	Alcohol	-0.283	0.015	0.756	0.281	0.189
3-Methylbutanal	Aldehyde	0.847	0.194	-0.121	0.235	0.236
Acetaldehyde	Aldehyde	-0.026	0.013	-0.018	-0.387	-0.292
2-Methylpropanal	Aldehyde	0.370	-0.292	0.034	0.502	-0.626
2-Methylbutanal	Aldehyde	0.408	-0.157	0.020	0.534	-0.634
Ethyl Acetate	Ester	-0.506	0.007	0.671	-0.018	-0.053
2-Hydroxyethyl propanoate	Ester	-0.551	-0.215	-0.590	0.222	0.135
Ethyl butanoate	Ester	0.832	-0.510	0.066	0.088	0.156
Ethyl hexanoate	Ester	0.750	-0.523	0.070	-0.053	0.185
Acetone	Ketone	0.074	0.863	0.256	0.118	-0.021
2-Heptanone	Ketone	-0.576	-0.062	-0.137	0.183	0.195
Diacetyl	Ketone	-0.638	-0.427	0.052	0.495	0.308
Acetoin	Ketone	-0.588	-0.199	-0.586	0.404	0.145
2-Butanone	Ketone	0.360	0.884	-0.119	0.224	0.057
2-Pentanone	Ketone	0.387	0.842	-0.117	0.192	0.106
2,3-Pentanedione	Ketone	0.765	-0.430	0.053	-0.136	0.300
3-Hydroxy-3-methyl-2-butanone	Ketone	0.102	-0.057	-0.002	-0.405	-0.007
Dimethyl sulfide	Sulfur compound	0.331	0.867	-0.117	0.226	0.045
Dimethyl disulfide	Sulfur compound	0.440	0.856	-0.120	0.183	0.090
Dimethyl trisulfide	Sulfur compound	0.424	0.513	-0.085	0.030	0.327
Methanethiol	Sulfur compound	0.390	0.879	-0.123	0.173	0.091
S-Methyl thioacetate	Sulfur compound	0.325	0.860	-0.115	0.227	0.036
1,2-Dimethoxyethane	Other	-0.329	0.029	0.881	0.216	0.137
Chloroform	Other	-0.565	-0.223	-0.608	0.315	0.189
Pyridine	Other	-0.432	-0.174	-0.476	0.299	0.181
Toluene	Other	-0.197	-0.090	-0.260	0.176	0.095
2,5-Dimethylpyrazine	Other	0.311	-0.178	0.016	-0.219	0.434

Volatile compounds data of the control and cheese inoculated with *Pseudoalteromonas* sp. TS-4-4 samples obtained from ripening tests are used for principal component analysis.
